# Modelling Cochlear Mechanics

**DOI:** 10.1155/2014/150637

**Published:** 2014-07-23

**Authors:** Guangjian Ni, Stephen J. Elliott, Mohammad Ayat, Paul D. Teal

**Affiliations:** ^1^Institute of Sound and Vibration Research, University of Southampton, Southampton SO17 1BJ, UK; ^2^School of Engineering and Computer Science, Victoria University of Wellington, P.O. Box 600, Wellington 6140, New Zealand

## Abstract

The cochlea plays a crucial role in mammal hearing. The basic function of the cochlea is to map sounds of different frequencies onto corresponding characteristic positions on the basilar membrane (BM). Sounds enter the fluid-filled cochlea and cause deflection of the BM due to pressure differences between the cochlear fluid chambers. These deflections travel along the cochlea, increasing in amplitude, until a frequency-dependent characteristic position and then decay away rapidly. The hair cells can detect these deflections and encode them as neural signals. Modelling the mechanics of the cochlea is of help in interpreting experimental observations and also can provide predictions of the results of experiments that cannot currently be performed due to technical limitations. This paper focuses on reviewing the numerical modelling of the mechanical and electrical processes in the cochlea, which include fluid coupling, micromechanics, the cochlear amplifier, nonlinearity, and electrical coupling.

## 1. Introduction

### 1.1. Scope of the Review

Models are useful tools to connect our understanding with physical observations. The mammalian cochlea is the organ that converts sound into neural coding and has extraordinary sensitivity and selectivity. It is important to understand the mechanisms of mammalian hearing not only because of the scientific challenges they present but also because such knowledge is helpful in diagnosing and potentially treating the multiple forms of hearing problems from which people suffer. Modelling the mechanics of the cochlea assists in this understanding by allowing assumptions about its functions to be verified, by comparing responses predicted by mathematical models with experimental observations. A cochlear model can be thought of as a tool with which to carry out “numerical experiments,” in which researchers can obtain or predict output response to different stimuli. These predictions can then be used to compare with experimental observations and hence help to refine and validate the model or even to provide a guide on measurements that cannot be performed in experiments due to technical limitations. The type of cochlear modelling undertaken also depends on the purpose of the study and the available data of the cochlea.

This review will focus on numerical modelling of the mechanical and electrical processes that lead to the vibrations of the BM, the cochlear amplifier, and other nonlinear behaviours, in the mammalian cochlea. Some classical cochlear models will be illustrated to give a physical insight into how the cochlea works. This is not to judge which model is the best but to review the progress of cochlear modelling work.

### 1.2. Anatomy of the Cochlea

The cochlea can be taken as a frequency analyser residing in the inner ear. The principal role of the cochlea is to transform the hair cell motions induced by the incoming sound wave into electrical signals. These electrical signals then travel as action potentials along the neural auditory pathway to structures in the brainstem for further processing. The whole transformation can be seen as a procedure of a real time spectral decomposition of the acoustic signal in producing a spatial frequency map in the cochlea. Mammalian auditory systems have the capability of detecting and analysing sounds over a wide range of frequency and intensity; for example, humans can hear sounds with frequencies from 20 Hz to 20 kHz and over an intensity range up to 120 decibels. This remarkable performance depends on mechanical and biophysical processes in the cochlea and the peripheral organ of hearing.

**Figure 1:**
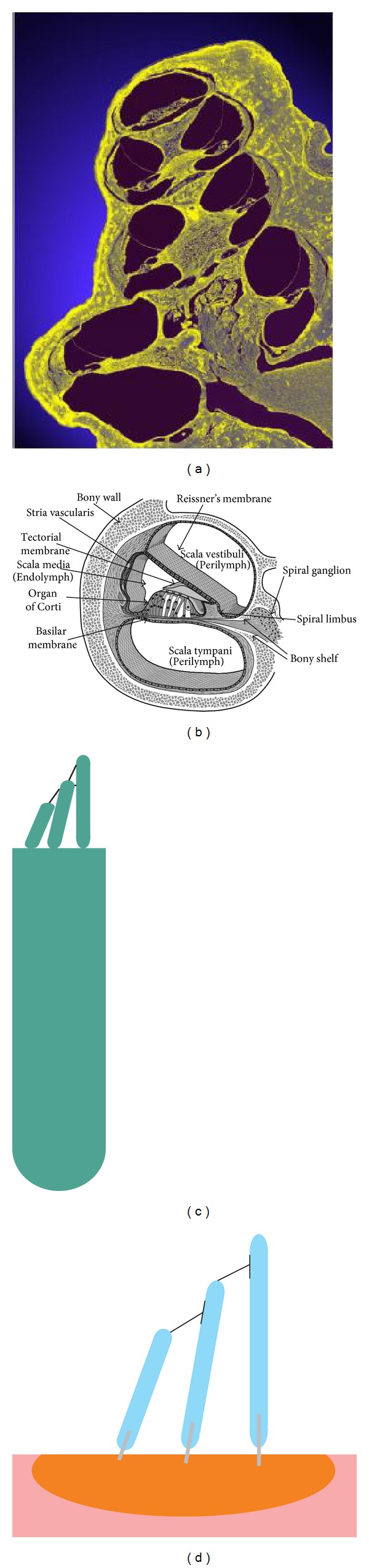
(a) A lateral view of the cochlear structure [41] (reprinted from American Journal of Otolaryngology, 33, Marinković et al., Cochlea and Other Spiral Forms in Nature and Art, 80–87, Copyright (2011), with permission from Elsevier). (b) The detailed structure of the OC [42] (with permission from author). (c) The structure of a hair cell. (d) Schematic drawing of the hair bundle.

The cochlea consists of a coiled labyrinth, like a snail, which is about 10 mm across and has about 2.5 turns in humans, embedded in the temporal base of the skull. It is filled with fluid and divided into three main fluid chambers, as described, for example, by Pickles [1], and shown in Figure 1(a).  Figure 1(b) shows that the scala vestibuli is at the top, which is separated from the scala media by a thin flexible partition called Reissner's membrane, and the scala media are separated from the scale tympani at the bottom by a rigid partition that includes a more flexible section called the basilar membrane.

Neither the coiling nor RM is believed to play a major role in the mechanics of the cochlea; the dynamics of which can thus be analysed in terms of two fluid chambers separated by the BM. The motion in the cochlea is driven by the middle ear via a flexible (oval) window at the basal end of the upper fluid chamber, and the pressure at the basal end of the lower fluid chamber is released by another flexible (round) window. It is thus the difference in pressure between the upper and lower fluid chambers that drives the BM. The OC sits on top of the BM and contains two types of hair cells, as shown in Figure 1(b). Each cross-section of the OC contains a single IHC, which converts the motion of the stereocilia into neural impulses that then pass up the auditory pathway into the brain. There are also three rows of OHCs within the OC that play a more active role in the dynamics of the cochlea. The individual stereocilia of a hair cell are arranged in a bundle, as shown in Figure 1(c). When this bundle is deflected towards the longest unit, the fine tip links that connect the individual stereocilium are put under tension and open gating channels that allow charged ions from the external fluid into the stereocilia and hence into the hair cells, as shown in Figure 1(d). The current due to this ionic flow generates a voltage within the hair cell, due to the electrical impedance of its membrane. In the IHC, it is this voltage, once it is above a certain threshold, which triggers the nerve impulses that send signals to the brain. The effect of this voltage on the OHCs is still being investigated in detail, but it is clear that it leads to expansion and contraction of the cell, which amplifies the motion in the OC at low levels.

This electromotility of the OHCs, as it is called, is due to a unique motor protein (Prestin) of the cell membrane that changes its shape when a voltage is applied, much like a piezoelectric actuator. The overall action of each OHC is thus to sense motion within the OC, via its stereocilia, to control the voltage within it, via the gating channels and capacitance, and to generate a response, via electromotility. There are about 12,000 OHCs in the human cochlea and they each act through this mechanism as local feedback controllers of vibration. It is surprising how this large number of locally acting feedback loops can act together to give a large and uniform amplification of the global response of the BM. It is also remarkable how quickly the OHCs can act, since they can respond at up to 20 kHz in humans and 200 kHz in dolphins and bats. This is much faster than muscle fibres, for example, which use a slower, climbing mechanism to achieve contraction. This climbing mechanism is still used within the stereocilia, however, to regulate the tension in the tip links and thus maintain the gating channels at the optimum point in their operating curves [2].

### 1.3. Cochlear Mechanics

As previously mentioned, the principal role of the cochlea is to transform the hair cell motion induced by the incoming sound wave into electrical signals. These electrical signals then travel as action potentials along the auditory pathway to structures in the brainstem for further processing. Carterette [3] summarized the history, from the ancient Greeks to modern day, of studies of auditory anatomy and function. He shows that at the early stages, the studies were mainly focusing on anatomy and identifying the major features of the auditory system like the eardrum, the cochlea, and bones of the middle ear. von Békésy [4] carried out pioneering work to reveal the waves in the cochlea extracted from human cadavers in the 1940s. He found that a travelling wave generated by a pure tone excitation propagated along the BM with wave amplitude gradually increased. After a peak at a specific location, where resonance occurs, the vibration decays quickly along the BM. The frequency of the input tone determines the location at which the peak occurs and this peak is more basal at high frequencies and more apical at low frequencies. This behaviour is one of the most critical evaluation criteria for cochlear models.

**Figure 2:**
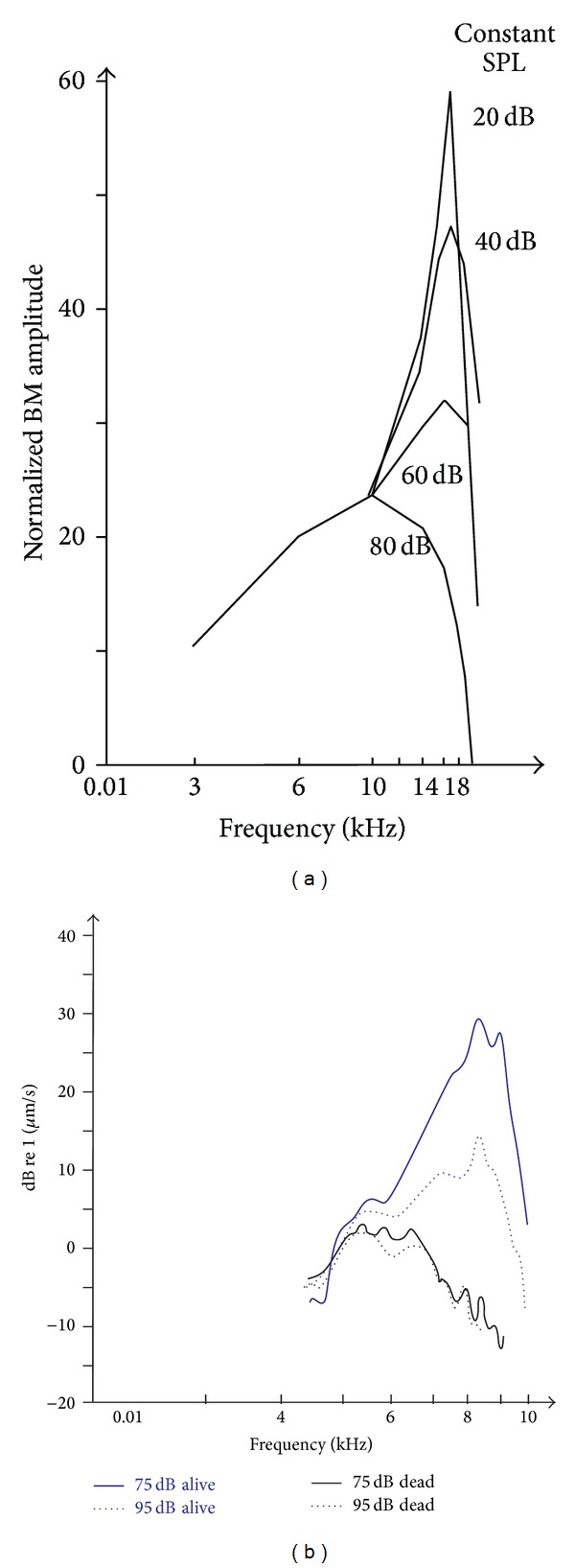
(a) The normalised BM amplitude at different sound pressure levels (SPL). All curves converge below 10 kHz, indicating linear response and equal gain, independent of the SPL. Measurements were performed using the Mössbauer technique in the basal turn of the guinea pig cochlea. Maximal response frequency is at about 17 kHz [43] (reprinted from Hearing Research, 22, Johnstone et al., Basilar Membrane Measurements and the Travelling Wave, 147–154, Copyright (1986), with permission from Elsevier). (b) Gain functions of the BM displacement measured in the basal turn of the chinchilla cochlea with laser Doppler velocimetry. Maximal response frequency is at about 8.5 kHz. Measurements are shown at two sound pressure levels, 75 and 95 dB, and in conditions of living and dead cochleas [44] (reprinted from Journal of Neuroscience, 11, Ruggero and Rich, Furosemide Alters Organ of Corti Mechanics: Evidence for Feedback of Outer Hair Cells upon the Basilar Membrane, 1057–1067, Copyright (1991), with permission from Copyright Clearance Centre).

The first finding related to the nonlinearity in the cochlea was back in 1971. Rhode [5] pointed out that the BM response to sinusoidal stimuli is less frequency selective for higher level stimuli. With the development of more refined measurement technologies, more and more evidence showed that the cochlea is active and nonlinear. The idea of active processes in the cochlea was first raised by Gold [6] and evidenced by Kemp [7] in the form of objective tinnitus and otoacoustic emissions. These active processes provide a frequency-sharpening mechanism. Lyon [8] and Mead [9] emphasized that the active processes function primarily as an automatic gain control, allowing the amplification of sounds that would otherwise be too weak to hear. The response of the BM in living ears was found to be different both qualitatively and quantitatively from that seen in dead ears. From Figure 2(b), the nonlinearity, as well as the sharp tuning behaviour, of the living cochlea is seen to be different from that of the dead one. In the living cochlea, the gain is higher at the lower stimulus level, but for the dead cochlea this gain difference disappears and the tuning becomes independent of the stimulus level providing evidence of a nonlinear active process. Other evidence of the active behaviour in the living cochlea is given by the detection of sound in the ear canal, due to spontaneous oscillations originating from the cochlea, retransmitted by the middle ear, in the absence of any excitation [10].

**Figure 3:**
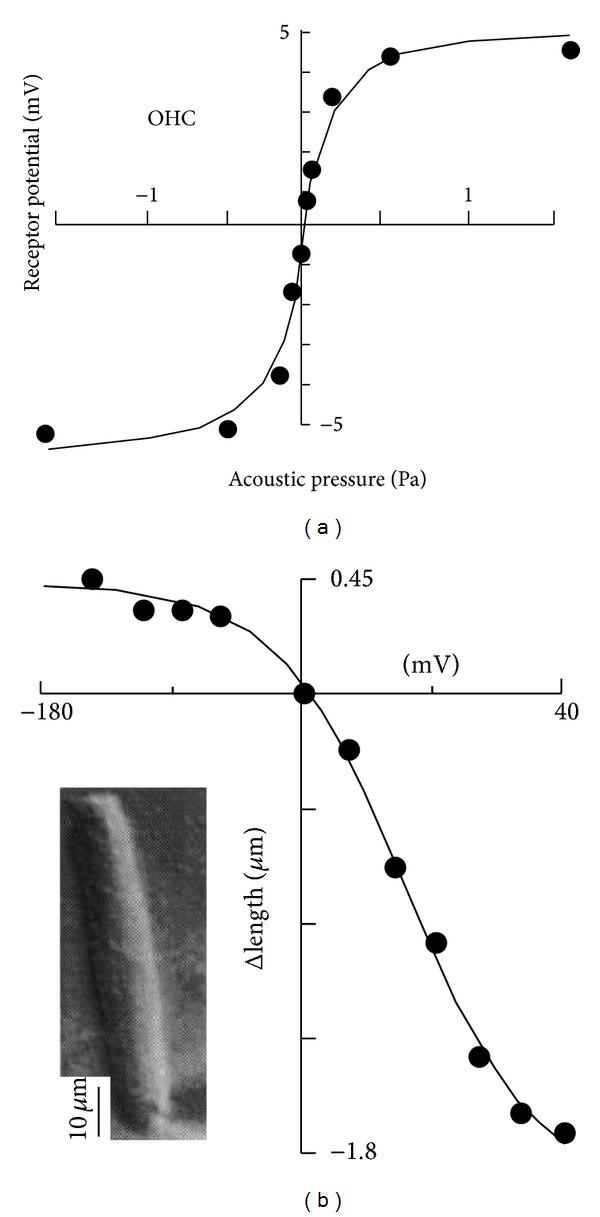
Saturating profile of outer hair cells. (a) The relation between acoustic pressure and outer hair cell receptor potential is S-shaped, saturating at high pressure levels [45] (reprinted from Hearing Research, 22, Russell et al., The responses of inner and outer hair cells in the basal turn of the guinea pig cochlea and in the mouse cochlea grown* in vitro*, 199–216, Copyright (1986), with permission from Elsevier). (b) Changes in the cell body length of an isolated outer hair cell in response to various transmembrane voltage steps are also S-shaped [46] (reprinted from Journal of Neuroscience, 12, Santos-Sacchi, On the Frequency Limit and Phase of Outer Hair Cell Motility: Effects of the Membrane Filter, 1906–1916, Copyright (1992), with permission from Copyright Clearance Centre). As can be seen, hyperpolarization elicited elongation, while depolarization caused contraction. Dots represent raw data. Solid line represents Boltzmann function. Insert represents outer hair cell.

It has been discovered that OHCs have a saturation property, which yields nonlinear responses. The relation measured between sound pressure and receptor voltage for OHCs shows a typical S-shape as depicted in Figure 3(a). In addition, the length change of the OHCs saturates with its transmembrane potential, as shown in Figure 3(b). One of the most significant nonlinear behaviours of the cochlea is high sound-level compression. Sound signals at low intensities are amplified in a frequency-selective manner at certain cochlear position, where the cochlea exhibits large gain, while high-level sound signals are barely amplified, where the cochlea exhibits small gain, as shown in Figure 2(a). Thus, the cochlear responses at the peak show compressive growth with input intensity. From an engineering point of view, the cochlea accomplishes automatic gain control, in which the gain of the cochlear amplifier becomes attenuated with increase in input intensity.

### 1.4. Levels of Detail in the Cochlear Model

**Figure 4:**
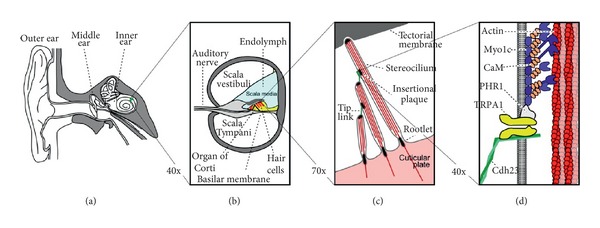
Illustrations of the structure of the inner ear at various levels of magnification. The position of the inner ear in the temporal bone is shown in (a). The cross-sectional structure within one turn of the cochlea is shown in (b) with the fluid chambers separated by the basilar membrane and the organ of Corti. The details of the bundle of stereocilia that protrude from the top of the hair cells within the organ of Corti are shown in (c). Finally (d) shows the molecular details of the myosin motors that maintain the tension in the tip links that connect the individual stereocilia within the bundle. The transduction channels (here labelled TRPA1) are now believed to reside at the bottom end of the tip link rather than the top [47] (reprinted from Neuron, 48, LeMasurier and Gillespie, Hair-Cell Mechanotransduction and Cochlear Amplification, 403–415, Copyright (2005), with permission from Elsevier).

One clear difference between cochlear models is the level of detail included in the models. The cochlea is a multiscale arrangement of different cellular and membranous components, whose dimensions vary from 10^−3^ m down to 10^−8^ m, as shown in Figure 4. In cochlear macromechanics, the vibration of one radial section of the CP is often simplified to BM movement only. In this way, the CP is often modelled as a series of independent segments, each of which represents a beam or plate strip with a predefined mode shape, yielding a relatively simple radial profile of vibration. In cochlear micromechanics, the vibrations of the different parts of the CP in relation to each other are modelled, as well as the detailed motions of the cellular structures within the OC. To achieve a reasonably complete understanding of cochlear function, the model should be able to explain how the vibrations of the cellular and membranous components of the CP result in deflections of the IHC stereocilia. Thus it is of immense interest to investigate the “micromechanics” of the cochlea, that is, how various sites of the OC, the BM, and the TM move in relation to each other, as shown in Figure 1(b).

The current models of the micromechanics of the OC often use a lumped-parameter representation of the BM, TM, and the structures into which the hair cells are embedded. The other way to study the micromechanics of the cochlea is using numerical methods such as the finite element method which is powerful in modelling complex structures. Determining the optimal complexity of a model is largely dependent on the modelling purpose and available (known) material properties. If the model is too simplistic, it will not embody the important processes of the real system. More details could be included if the needed geometry of the anatomical structure and material properties are available. The analysis time for a system may be inevitably increased with increase of system complexity. Lim and Steele [11] adopted a hybrid WKB-numeric solution for their nonlinear active cochlear model, in which the WKB method was used in the short wave region and numerical Runge-Kutta method was used in the long-wave region, to keep computation fast and efficient.

## 2. Types of Cochlear Models

Compared to reality, cochlear models may be incredibly simplified, but these crude models can still reflect important components of how the real organ works. The motivations of modelling the cochlea are to represent, within one framework, the results from a large variety of experiments and to explain the functions of the hearing system. In principle, models should also be testable by providing predictions of experiments that have yet to be done. Cochlear models have been formulated and constructed in various forms. These models are concerned with mechanical structures built up with structural elements like plates or beams coupled with fluid [12] or electrical networks [13] consisting of inductors, resistances, capacitors, diodes, and amplifiers. After construction, these structures can be put into mathematical form and then be solved numerically.

Models of cochlear mechanics are constructed to replicate basic physiological properties, such as the fundamental and harmonic cochlear responses to a single tone stimulus and then applied to interpret more complex observations and develop valid experimental hypotheses. For example, cochlear modelling was used by Helmholtz (1877) to explore perception of tones and by Gold and Pumphrey [14] to interpret the sharp tuning observed in the cochlea and to predict otoacoustic emissions. More recently models have been used to demonstrate that a cochlear amplifier mechanism is necessary to explain the sharply tuned response of the BM to single tone stimulation [15]. Many different types of cochlear model have been proposed including physical models, constructed either from plastic and metal materials or electrical networks [16–18] and computed mechanical models [12, 19–22]. Such models, where the cochlea is split into finite segments in the longitudinal direction, have varying numbers of degrees of freedom ranging from 1 to over 1000 per slice [23, 24]. Early cochlear models were designed to simulate only the amplitude and phase of linear, passive response of the cochlea to single tone stimulation [25–29]. Models then progressed to incorporate an active process and nonlinearity [19, 30–32]. The nonlinear models were either solved in the frequency domain using iterative or perturbation techniques [33–35] or in the time domain [22, 36–40].

### 2.1. Traveling Waves in the Cochlea

Most descriptions of the mechanical response of the cochlea involve the forward propagation of a single, “slow,” wave [26, 49]. This wave is generated by an interaction between the inertia of the fluid in the chambers of the cochlea and the stiffness of the BM and can be reproduced using simple one-dimensional box models [12]. At low sound pressure levels the amplitude of this wave is amplified by a number of active processes within the OC, but the basic description of slow wave propagation is valid even when the cochlea is passive and also for high sound pressure levels. Since the properties of the cochlea, particularly the BM stiffness, vary along its length, the properties of this slow wave are position-dependent when excited at a given driving frequency. These properties can be characterised at each position along the cochlea by a complex wavenumber; the real part determines the wave speed and the imaginary part determines the spatial attenuation of the wave.

If the wavenumber distribution along the cochlea can be calculated from a model, or inferred using an inverse method from measurements [15], the mechanical response of the cochlea can then be calculated using the WKB method [26]. The WKB method has a number of inherent assumptions, however, such as that the wave is only travelling in one direction. This implies that no backward travelling wave is generated by the normal hearing function of the cochlea, even though such waves are believed to be responsible for other phenomena such as otoacoustic emissions, for example. Another assumption is that the wavenumber does not vary too rapidly with position, as compared with the wavelength [49], although this assumption appears to limit the applicability of the WKB method in cochlear modelling less than one would expect [50]. Zwislocki [51, 52] predicted the delay of the travelling wave to accumulate with increasing distance from the stapes. Steele [53] firstly adopted the WKB method to solve cochlear mechanical problems and found closed-form solutions for a 1D cochlear model. Zweig et al. [26] found the closed-form WKB solutions for a 1D long-wave model in 1976. Steele et al. also extended the WKB method to solve 2D [54] and 3D [23, 55] cochlear problems. de Boer and Viergever [49, 56] further developed the WKB approach for cochlear mechanics. The WKB solutions for the 2D and 3D cochlear model showed good agreement with more detailed numerical solutions, except for the region just beyond the BM response peak, which was suggested to be due to the nonuniqueness of the complex WKB wavenumber in 2D and 3D models [56]. Elliott et al. [57, 58] applied the wave finite element method to decompose the full BM responses of both passive and active cochlear models in terms of wave components. They found besides the conversional slow wave, an evanescent, higher-order fluid wave starts to make a significant contribution to the BM response in the region apical to the peak location.

In the travelling wave theory, the “slow” wave propagates on the BM from base to apex [4] and the energy incoming from the stapes is transported in the cochlea primarily via pressure waves in the fluid, since the longitudinal coupling in the BM is believed to be very weak. von Békésy [59] first observed the traveling wave caused by a pure tone input in a cadaver cochlea, which carries displacement patterns propagating along the BM. The wave amplitude increases gradually to a peak at a characteristic location along the BM, after which it decays rapidly. The characteristic location depends on the driving frequency; for example, the peak is close to the stapes at high frequencies and further towards the apex at lower frequencies. This “place principle” is a crucial mechanism of frequency analysis in the cochlea and is caused primarily by changes in the stiffness of the BM.

In a general way, once we know the wavenumber *k*, the displacement of the BM produced by a pure tone can be expressed using the WKB approximation [56] as

(1) $$\begin{array}{c} w\left(x,t\right)=Ak{\left(x,\omega \right)}^{3/2}e^{i[\omega t-\phi (x)]}, \end{array}$$

where *ϕ*(*x*) = ∫_0_^*x*^ *k*(*x*′, *ω*)*dx*′ denotes the integral of the accumulating phase shift and gains or losses as the wave propagates along the cochlea, *x*′ is a dummy integration variable, factor *A* is the wave amplitude at the base, and *ω* = 2*πf* is the driving frequency. The additional *k*(*x*, *ω*)^3/2^ term is necessary for conservation of energy when the wavenumber changes with *x*.

**Figure 5:**
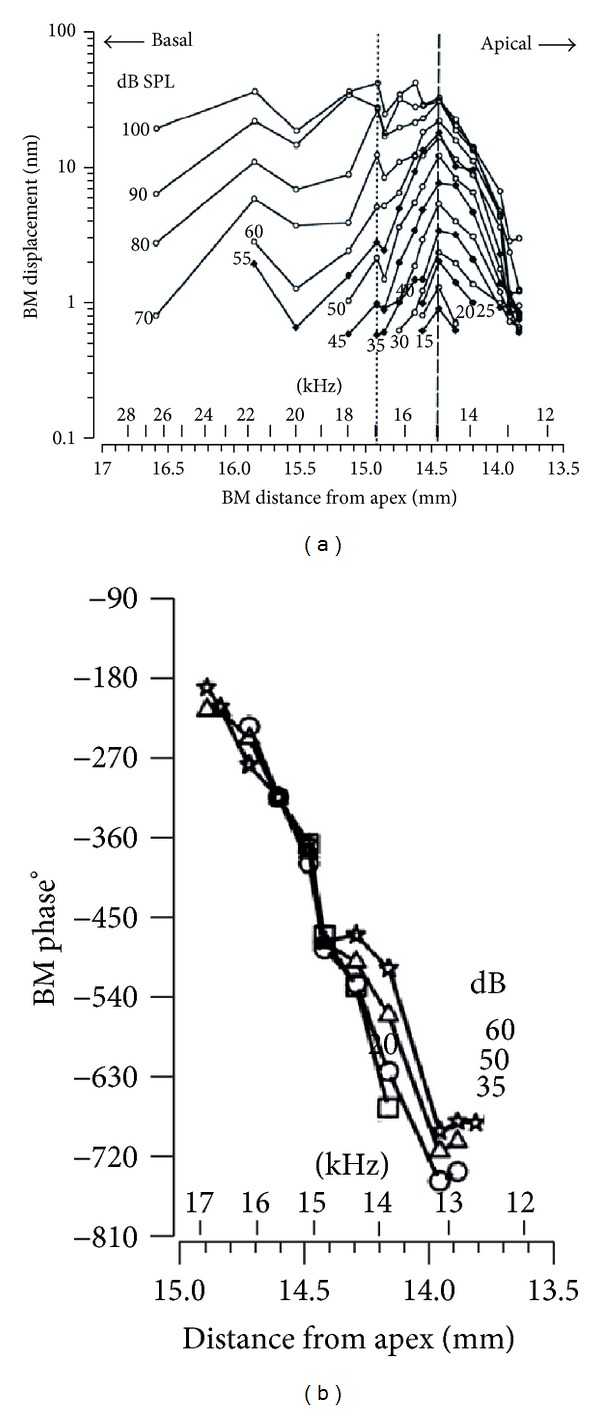
BM displacement (a) magnitude and (b) phase distribution along the cochlear longitudinal direction, plotted as a function of distance from the apex, in response to a 15 kHz tone over a range of intensities from 15 to 60 dB SPL [48] (reprinted from PNAS, 94, Russell and Nilsen, The Location of the Cochlear Amplifier: Spatial Representation of a Single Tone on the Guinea Pig Basilar Membrane, 2660–2664, Copyright (1997) National Academy of Sciences, USA).

From the experimental point of view, studies of the travelling wave were based solely on measurements of BM motion [43]. Direct demonstrations of the traveling wave were obtained by measuring the phase accumulation of the BM in response to identical stimuli [60]. Russell and Nilsen [48] applied several 15 kHz tones with different intensities at the base of a guinea pig cochlea to measure the BM displacement and phase lags expressed as a function of distance from the stapes. It can be seen from Figure 5 that the phase accumulation between the CF site and 1 mm basal to the CF is about 1.5 cycles for 35 dB tones, indicating a wavelength at CF of about 0.67 mm and a wave velocity of about 10 m/s [60]. Generally, the travelling wave is gradually slowing down with a decreasing wavelength from the basal end until it approaches the CF site and then decays rapidly.

**Figure 6:**
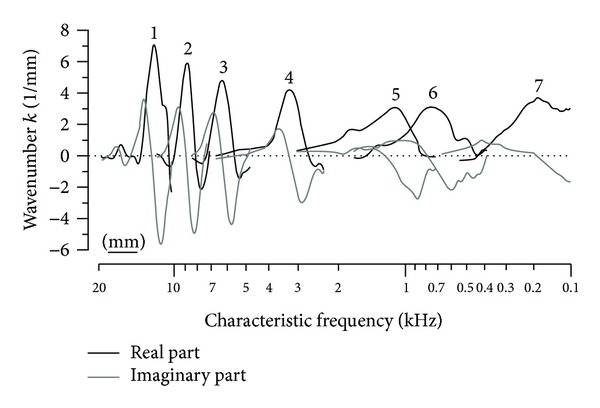
The distribution of the real (black lines) and imaginary (grey lines) parts of the wavenumber inferred from measurements of the BM frequency response at seven positions along the length of the cochlea using an inversion procedure [15] (reprinted with permission from Journal of the Acoustical Society of America, 122, Shera, Laser Amplification with a Twist: Traveling-Wave Propagation and Gain Functions from throughout the Cochlea, 2738–2758, Copyright (2007), Acoustic Society of America).

Olson [61] developed an elegant way to measure intracochlear pressure close to the cochlear partition. The fluid pressure is a fundamental element of the travelling wave theory. The observation of the slow pressure waves shows consistency with those from BM motion and the observed phase lags of the slow pressure wave are consistent with those of BM vibration. Shera [15] proposed an inverse method for using the experimentally obtained BM velocity transfer function at a location along the* in vivo* cochlea in the frequency domain to calculate the propagation and gain functions. He then went on to reconstruct the BM velocity distribution in the spatial domain to test the theory. This method gives strong evidence for travelling wave amplification in the mammalian cochlea based on BM velocity measurements, which are the real and imaginary parts of the complex wavenumber, as shown in Figure 6.

**Figure 7:**
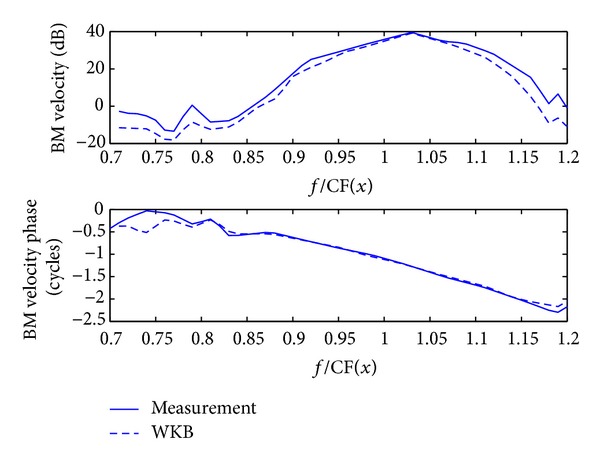
The BM velocity distribution reconstructed from the derived wavenumber using the WKB approximation. The reconstructed response (dashed lines), obtained using the WKB approximation, shows good agreement with that from measurement (solid lines) (reproduced with permission from Journal of the Acoustical Society of America, 122, Shera, Laser Amplification with a Twist: Traveling-Wave Propagation and Gain Functions from throughout the Cochlea, 2738–2758, Copyright (2007), Acoustic Society of America).

The method can also be used to reconstruct the BM velocity distribution in combination with the WKB approach, (1).  Figure 7 shows good agreement between the original measured BM magnitude and phase distributions and those reconstructed from the derived wavenumber using the WKB approximation [15]. This gives both strong theoretical and practical evidence to support the travelling wave theory in the cochlear mechanism. Since these measurements were taken on an active cochlea, the imaginary part of the wavenumber is not entirely negative, indicating that the active processes are amplifying the wave at positions just before it reaches its peak. Apart from this aspect the distributions of the real and imaginary wavenumbers are similar to those predicted from the simple analytic passive models [12, 23].

#### 2.1.1. Box Model of the Cochlea

**Figure 8:**
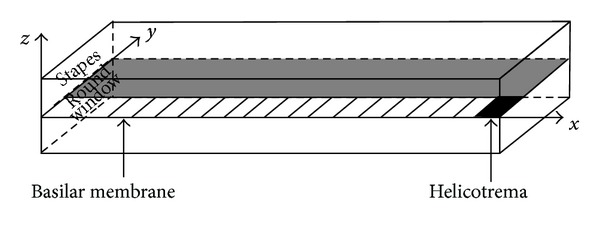
A simple box model of the cochlea consists of two fluid chambers separated by the BM. The longitudinal coordinate, *x*, goes from the left, base, to the right, apex, and an external pressure is applied on the left side (by the stapes) to represent vibration transmitted from the ossicles. The two fluid chambers, SV and ST, are separated by a flexible BM, which occupies part of the cochlear partition width, and connect to each other at the end of the model via the helicotrema, where the pressure difference between the two chambers is zero.

The real structure of the cochlea and the components within it are very complicated [62, 63]. In order to replicate the basic functions of the cochlea, the real structure of the cochlea has to be simplified to be practical for numerical modelling. Generally, the coiled cochlea is represented by a straight sandwich structure, box model, with two fluid chambers, SV and ST, separated by the BM. In order to describe the box model with mathematical formulae, assumptions and boundary conditions are needed to make the model numerically available and physically meaningful. The assumptions below are for the box model, as shown in Figure 8, and may not hold for models used for specific studies, geometrical nonuniformity or CP longitudinal coupling, for example.

1. The cochlear walls are immobile and rigid indicating the pressure gradient is zero on the walls [64].
2. The effect of “fluid ducts” can be neglected [64, 65].
3. The spiral shape of the cochlea is straightened out. This may lose some information in the apical region of the model [66, 67], where the cochlear curvature is greatest, but this is neglected as there is limited physiological data available for the apical region.
4. Reissner's membrane is neglected as it is acoustically transparent [68, 69].
5. The two cochlear channels have equal cross-sectional area and shape, so pressures of upper, SV, and lower, ST, fluid chambers are equal with opposite sign [12]. This assumption is not necessary for those box models with varying geometry along its length [70]. The cross-sectional area of the chambers is assumed to be rectangular, although de Boer [71] has shown that similar results are obtained if the cross-section is assumed to be semicircular. The effective height of the chambers (the ratio of the cross-sectional area to the width of the chamber) is assumed to be constant and neglect any variation with distance from the base (this assumption is only applicable for a uniform 1D box model).
6. The boundary condition at the helicotrema is assumed to be pressure release; that is, the pressure difference is equal to zero. This can alternatively be more accurately modelled involving friction terms [72].
7. The cochlear fluids have negligible viscosity, so that only the CP dissipates energy [12]. This is because cochlear input impedance is not significantly affected by the introduction of the fluid viscosity for frequencies greater than 500 Hz [73, 74]. The cochlear fluids and CP are incompressible [12].
8. There is no structural longitudinal coupling along the CP and elements along the CP interact through fluid coupling only [12].

In many box models of the cochlea [12, 52, 75], the cochlear partition is defined as a unit that interacts with the cochlear fluids. Although this assumption neglects individual movements of elements inside, it can reasonably well approximate cochlear macromechanics. In such models, the motion of the CP is often referred to as that of the BM, since the BM is believed to dominate the mechanics of the OC passively [4].

#### 2.1.2. Elemental Cochlear Model

It is computationally convenient to divide a continuous system into a number of discrete elements, which may be taken as an accurate representation of the continuous system if there are at least six elements within the shortest wavelength present, which is a condition commonly used in finite element analysis [76]. The linear coupled behaviour of the cochlear dynamics can then be represented by matrix representations of two separate phenomena. First, the way that the pressure distribution is determined by the fluid coupling within the cochlear chambers when driven by the BM velocity, and second, the way in which the BM dynamics respond to the imposed pressure distribution. This kind of elemental model was used, for example, by Neely and Kim [19], to simulate an early model of the active cochlea, and has been used by many authors since then.

The analysis can be generalised to the case in which the radial BM velocity is the sum of a number of such modes [77]. Here, for the purpose of illustration, a single shape is assumed for the BM radial velocity profile, since the fluid coupling is relatively insensitive to the exact form of the radial BM velocity distribution. The radial variation of BM velocity over the width of the CP, *W*, is assumed to be proportional to a single mode shape, *ψ*(*y*), which is independent of the distribution of the pressure acting upon it but dependent on the boundary conditions assumed for the BM [78].

**Figure 9:**
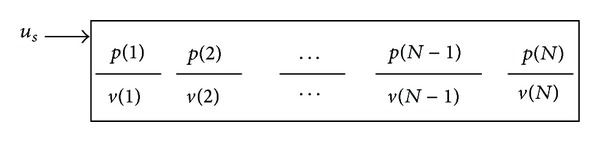
The discrete approximation for a straightened cochlear box model.

The single longitudinal variables for the modal pressure difference and the modal BM velocity are spatially sampled as finely as required, dividing the cochlea into *N* segments. At a single frequency, the vectors of complex modal pressure differences and modal BM velocities, **p** and **v**, can be written as [70]

(2) $$\begin{array}{c} p={\left[p\left(1\right),p\left(2\right),\ldots ,p\left(N\right)\right]}^{T},\\ v={\left[v\left(1\right),v\left(2\right),\ldots ,v\left(N\right)\right]}^{T}; \end{array}$$

the elements of which are shown in Figure 9.

The BM, however, is assumed only to extend from element 2 to element *N* − 1. Element 1 is used to account for the effect of the stapes velocity, shown as *u*_*s*_ in Figure 9. The final element, *N*, is used to account for the behaviour of the helicotrema. With the stapes velocity set to zero, the vector of pressures due to the vector of BM velocities can be written as

(3) $$\begin{array}{c} p=Z_{\text{FC}}v, \end{array}$$

where **Z**_FC_ is a matrix of the impedances due to the fluid coupling. Analysis of the form of the elements in this fluid coupling matrix is an important part of this type of modelling. Similarly, the vector of BM velocities can be written as

(4) $$\begin{array}{c} v=v_{\text{s}}-Y_{\text{BM}}p, \end{array}$$

where **v**_s_ is vector with first element the stapes velocity and **Y**_BM_ is a matrix of the BM admittances. The first and last diagonal elements are zero, since the BM only extends from element 2 to element *N* − 1. If the BM reacts only locally, then **Y**_BM_ is a diagonal matrix. Substituting (3) into (4) gives the vector of BM velocities as

(5) $$\begin{array}{c} v={\left[I+Y_{\text{BM}}Z_{\text{FC}}\right]}^{-1}v_{\text{s}}. \end{array}$$

The total pressure vector due to both stapes motion and motion of the BM can be written, using linear superposition, as

(6) $$\begin{array}{c} p=p_{s}+Z_{\text{FC}}v, \end{array}$$

where **p**_*s*_ is the vector of pressures due to the stapes velocity. Combining (5) and (6) gives

(7) $$\begin{array}{c} p={\left[I+Z_{\text{FC}}Y_{\text{BM}}\right]}^{-1}p_{s}. \end{array}$$

An advantage of this discrete formulation is that complicated geometries need to be analysed only once to determine the elements of **Z**_FC_, using the finite element method for example, [70], and (5) then provides a very simple method of calculating the coupled responses, for a variety of models, with a coiled cochlea, for example, [79], of BM dynamics.

The frequency to place mapping that occurs within the cochlea can be described in terms of the propagation of a dispersive travelling wave within it. This wave motion involves interaction between the inertia of the fluid chambers and the stiffness of the basilar membrane. It occurs even for excitation of the cochlea at high sound pressures, for which the active processes within the outer hair cells are saturated and do not contribute significantly to the dynamics. The fundamental wave behaviour can thus be understood in the passive cochlea, in which the feedback loops created by the outer hair cells are ignored. In a simple one-dimensional “box model” for the uncoiled cochlea, as shown in Figure 8, the velocity of the BM at a longitudinal position *x* and a frequency of *ω*, *v*(*x*, *ω*) depends only on the complex pressure difference between the fluid chambers at the same position *p*(*x*, *ω*), so that

(8) $$\begin{array}{c} v\left(x,\omega \right)=-Y_{\text{BM}}\left(x,\omega \right)p\left(x,\omega \right), \end{array}$$

where *Y*_BM_(*x*, *ω*) is the mechanical admittance, per unit area, of the basilar membrane, and the negative sign comes from defining *v*(*x*, *ω*) upwards, but *p*(*x*, *ω*) is positive with a greater pressure in the upper chamber. The fluid in the cochlea is assumed to be incompressible, since the cochlear length is much smaller than the wavelength of compressional waves in the fluid and also inviscid, since the height of the fluid chamber is much greater than the viscous boundary layer thickness, and damping is mainly introduced by the BM dynamics. The pressure is assumed to be uniform across each cross-section and the conservation of fluid mass and momentum can be used to derive the governing equation for one-dimensional fluid flow in the chambers, as described, for example, by de Boer [12], as

(9) $$\begin{array}{c} \frac{\partial^{2}p\left(x\right)}{\partial x^{2}}=-\frac{2i\omega \rho }{h}v\left(x\right), \end{array}$$

where *ρ* is the fluid density and *h* is the effective height of the fluid chambers, which is equal to the physical height of the fluid chamber in the 1D cochlear model. Substituting (8) into (9) gives the second-order wave equation

(10) $$\begin{array}{c} \frac{\partial^{2}p\left(x,\omega \right)}{\partial x^{2}}-k^{2}\left(x,\omega \right)p\left(x,\omega \right)=0, \end{array}$$

where the position and frequency-dependent wavenumber is given by

(11) $$\begin{array}{c} k\left(x,\omega \right)=\pm \sqrt{\frac{-2i\omega \rho }{h}Y_{\text{BM}}\left(x,\omega \right)}. \end{array}$$

The admittance of this single-degree-of-freedom model of the passive BM can be written as

(12) $$\begin{array}{c} Y_{\text{BM}}\left(x,\omega \right)=\frac{i\omega }{i\omega r\left(x\right)-\omega^{2}m\left(x\right)+s\left(x\right)}, \end{array}$$

where *m*(*x*), *s*(*x*), and *r*(*x*) are the effective mass, stiffness, and damping, per unit area, of the BM at position *x*. The distribution of natural frequencies, *ω*_*n*_(*x*), illustrated in Figure 11, can be assumed to be entirely due to the longitudinal variation of stiffness. The distribution of natural frequencies along the cochlea is approximately exponential so that

(13) $$\begin{array}{c} \omega_{n}\left(x\right)=\omega_{B}e^{-x/l}, \end{array}$$

when *l* is a characteristic length, taken here to be 7 mm, and *ω*_*B*_ is taken as 2*π* times 20 kHz for the human cochlea. The distribution of BM stiffness is then given by

(14) $$\begin{array}{c} s\left(x\right)=\omega_{n}^{2}\left(x\right)m_{0}=\omega_{B}^{2}m_{0}e^{-2x/l}. \end{array}$$

The distribution of the mechanical resistance, when a constant damping ratio, *ζ*_0_, is assumed along the BM, is then

(15) $$\begin{array}{c} r\left(x\right)=2\zeta_{0}m_{0}\omega_{n}^{}\left(x\right)=2\zeta_{0}m_{0}\omega_{B}^{}e^{-x/l}. \end{array}$$

**Figure 10:**
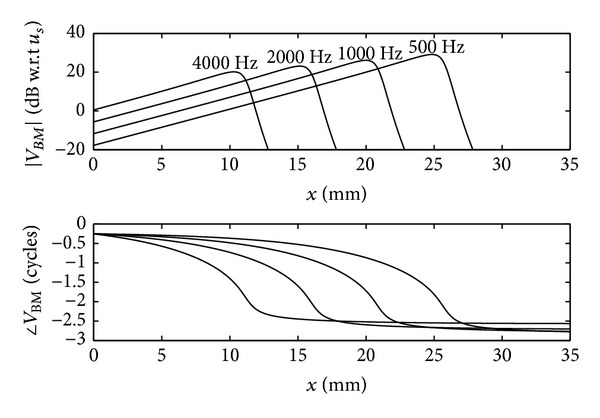
Simulations of the distribution of the magnitude and phase (plot with respect to the velocity at the stapes, *u*_S_) of the complex basilar membrane velocity along the length of the passive cochlea when excited by pure tones at different frequencies.

Since the wavenumber varies with position and frequency, conventional solutions to the wave equation in (10), for homogeneous systems, cannot be used. Provided the wavenumber does not change too rapidly compared with the wave length, however, an approximate global solution for *v*(*x*, *ω*) can still be obtained using the WKB method [26] as

(16) $$\begin{array}{c} v\left(x\right)=\frac{Ah}{2i\omega \rho }k{\left(x\right)}^{3/2}e^{-i\phi (x)},\\ v\left(x\right)=-Y_{\text{BM}}\left(x\right)\frac{A}{\sqrt{k\left(x\right)}}e^{-i\phi (x)}, \end{array}$$

where *A* is the amplitude, due to the driving velocity from the middle ear. It is found that, to a very good approximation, only a forward travelling wave exists in the cochlea, since this is almost perfectly absorbed as it travels along the cochlea, thus ensuring an optimum transfer of power from the middle ear.  Figure 10 shows the magnitude and phase of the BM velocity as a function of position along the cochlea, for four different driving frequencies, using the wavenumber distribution given by (11) for the passive BM. The phase is plotted in cycles, as is customary in the hearing literature, which, perhaps, should be adapted more widely, since it has more immediate physical significance than radians or degrees. One of the main features of the BM velocity distribution in Figure 10 is that they peak at different places for different excitation frequencies, providing a “tonotopic” distribution of frequency.

### 2.2. Lumped-Parameter Models

**Figure 11:**
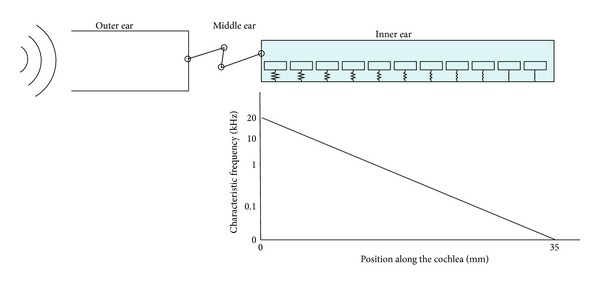
Idealised representation of the outer, middle, and inner ear, showing the basilar membrane in the inner ear as a series of mass-spring-damper systems distributed down the cochlea coupled together via the fluid shown in blue, together with the distribution of the natural frequencies of these single-degree-of-freedom systems.

The lumped-parameter model of the cochlea is a simplification of the OC. In this kind of model, the properties of the spatially distributed OC are represented by a topology consisting of discrete entities (masses, dampers and springs) that approximate the dynamic behaviour of the OC under certain assumptions. From a mathematical point of view, the dynamic behaviour of the OC can be described by a finite number of ordinary differential equations with a finite number of parameters. Mechanically, every component in the lumped-parameter model is taken as a rigid body and the connection between each rigid body takes place via springs and dampers. The model can be divided into a finite number of segments in the longitudinal direction with each individual segment having a unique characteristic resonant frequency, decreasing from 20 kHz, at the base, in the human, to about 200 Hz at the apex over the 35 mm BM length, as shown in Figure 11.

Various lumped-parameter models of the OC have been developed by researchers. The simplest one only contains one-degree-of-freedom, in which the TM is assumed only to move with the same motion as the BM. Allen [28] derived the relationship between the transverse motion of the BM and the shearing motion experienced by the OHC stereocilia. In his model, the TM is assumed only to rotate with the same angular movement as the BM. If the TM is allowed to move radially, the OC can be expressed by a two-degree-of-freedom model, in which the BM and the TM are assumed to move only in a single direction. It is also possible to apply the active force generated by the OHC on the model, as suggested by Neely and Kim [19], although it is difficult to physically justify what structure this force on the BM reacts off. An alternative active model is one in which the force is assumed to act across a very stiff OC, resulting in an active displacement, as in the model of Neely [80]. More detailed lumped-parameter micromechanical models have been proposed that have three degrees of freedom [32, 75] or even more.

An advantage of such lumped-parameter models, however, is that the conditions for stability, which is not guaranteed in an active model and can otherwise lead to misleading results, can be formulated using a state space representation [22]. It is also possible to use this representation to incorporate nonlinearity into the cochlear amplifier, which leads to compression of the dynamic range and many forms of otoacoustic emission or distortion products [34]. In the active cochlea, at least one extra mass has to be included in order to create a higher-order resonant system to replicate the greater frequency selectivity of the active cochlea.

### 2.3. Finite Element Models

Although the finite element cochlear model is an elemental representation of the real continuous cochlea, the flexibility of the finite elements allows the possibility of considering more detailed and complicated cochlear structure than in the elemental model above. In many areas, the finite element analysis is a key and indispensable technology in the modelling and simulation procedures. However, a good understanding of physical, mathematical, and computational modelling plays an important role in utilizing these advantages of the finite element method.

A finite element version of the cochlear box model can be obtained by dividing its length into *N*_*x*_ elements, in the *x* direction, and each fluid chamber into a *N*_*y*_ × *N*_*z*_ grid of hexahedral elements, in the *y* × *z* directions. Using symmetry it is only necessary to include a single fluid chamber in the numerical model. The BM within each of the *N*_*x*_ elements can be modelled as *N*_*y*_ thin plate (beam) elements, with no longitudinal coupling between each other. Each plate thus vibrates independently in the absence of the fluid and provides a locally reacting model of the BM. If the motion of the plate elements is represented by the vector **w**, then their dynamics can be written in the matrix form as

(17) $$\begin{array}{c} M\ddot{w}+Kw=Sp, \end{array}$$

where **M** and **K** are the mass and stiffness matrices for the plate, $\ddot{w}$ represents ∂^2^ **w**/∂*t* ^2^, and **p** is the vector of pressures in elements of the fluid chamber, which drive the plate via the coupling matrix **S**.

The dynamic response of the fluid can also be represented in finite element form [76] as

(18) $$\begin{array}{c} Q\ddot{p}+Hp=-\rho_{f}R\ddot{w}+q, \end{array}$$

where **Q** and **H** are acoustic mass and stiffness matrices, **q** is proportional to the external volume velocity due to the motion of the stapes, *ρ*_*f*_ is the fluid density, and **R** = **S** ^*T*^ denotes how the pressure is driven by the displacement of the plate elements. For the coupled system these two equations can be combined to give

(19) $$\begin{array}{c} \left[\begin{array}{cc} M & 0\\ \rho_{f}R & Q \end{array}\right]\left[\begin{array}{c} \ddot{w}\\ \ddot{p} \end{array}\right]+\left[\begin{array}{cc} K & -S\\ 0 & H \end{array}\right]\left[\begin{array}{c} w\\ p \end{array}\right]=\left[\begin{array}{c} 0\\ q \end{array}\right]. \end{array}$$

For a single frequency excitation, proportional to *e* ^*iωt*^,

(20) $$\begin{array}{c} \left[\begin{array}{cc} K-\omega^{2}M & -S\\ -\omega^{2}\rho_{f}R & H-\omega^{2}Q \end{array}\right]\left[\begin{array}{c} w\\ p \end{array}\right]=\left[\begin{array}{c} 0\\ q \end{array}\right], \end{array}$$

where damping can now be incorporated by using complex elements in the stiffness matrix.

Finite element techniques have also been applied to problems associated with cochlear micromechanics, including the motion of the hair cell stereociliary bundle [81] and the stiffness of individual OHCs [82]. They have also been used in complete cochlear models, with very simple representations of the OC, to investigate gross fluid motion both in two dimensions [83] and three dimensions [84]. Another study has modelled the OC with high structural accuracy and included nonlinear behaviour [85] within a short (60 *μ*m) section of the cochlea, but fluid-structure interactions were not included.

**Figure 12:**
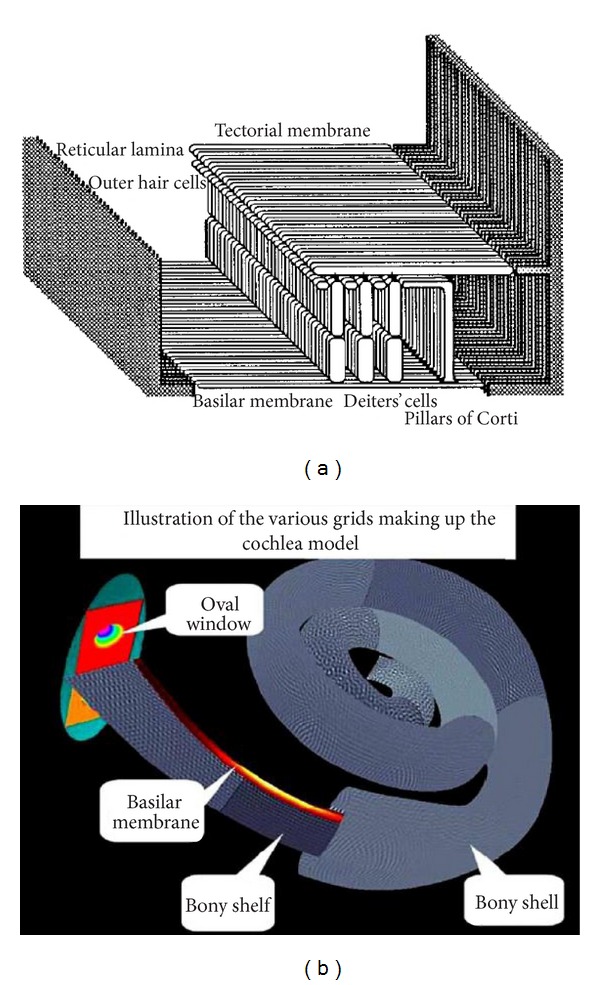
(a) An oblique view of a small section of the cochlear partition in the 3D FE modeling technique [86] (reprinted with permission from Journal of the Acoustical Society of America, 99, Kolston and Ashmore, Finite Element Micromechanical Modeling of the Cochlea in Three Dimensions, 455–467, Copyright (1996), Acoustic Society of America). (b) FE models of the cochlea constructed by Givelberg and Bunn [87]. In this view, several parts of the outer shell are removed in order to expose the cochlear partition consisting of the narrow basilar membrane and the bony shelf. The round window is located directly below the oval window and in this picture it is partially obscured by the cochlear partition (reprinted from Journal of Computational Physics, 191, Givelberg and Bunn, A Comprehensive Three-Dimensional Model of the Cochlea, 377–391, Copyright (2003), with permission from Elsevier).

Kolston and Ashmore [86] applied a 3D finite element network to build a 3D cochlear model, as shown in Figure 12(a), with individual cellular and membrane components of the OC being embedded within the fluid in their real biological positions and then solving the problem using the conjugate gradient method. The main new feature of the method is that it allows individual cellular and membrane components of the OC to be embedded within the model fluid in their true structural positions, with connections to neighbouring elements reflecting anatomical geometry. In spite of the large size of the resulting model, it has been implemented on an inexpensive computer and solved within acceptable time periods. They presented the results obtained from a small number of simulations suggesting that both the TM radial stiffness and especially the Deiters' cell axial stiffness play a crucial role in the OHC-BM feedback loop.

Givelberg et al. [87, 88] developed a detailed 3D computational model of the human cochlea, which was built based on geometry obtained from physical measurements, as shown in Figure 12(b). The model consists of the BM, spiral bony shelf, the tubular walls of the SV and ST, semielliptical walls sealing the cochlear canal, the oval window, and the round window membranes. The immersed boundary method, which is a general numerical method for modelling an elastic material immersed in a viscous incompressible fluid [89], was used to calculate the fluid-structure interactions produced in response to incoming sound waves. They used large shared memory parallel computers to run several large scale simulations. They observed a travelling wave propagating from the stapes to the helicotrema. The amplitude of the wave is gradually increasing to a peak at a characteristic location along the BM. The speed of the wave is sharply reduced as it propagates further along the BM after the peak. The higher the value of input frequency is, the closer the peak is to the base. Those observations are similar to experiments qualitatively, but this kind of comprehensive numerical model is computationally expensive.

**Figure 13:**
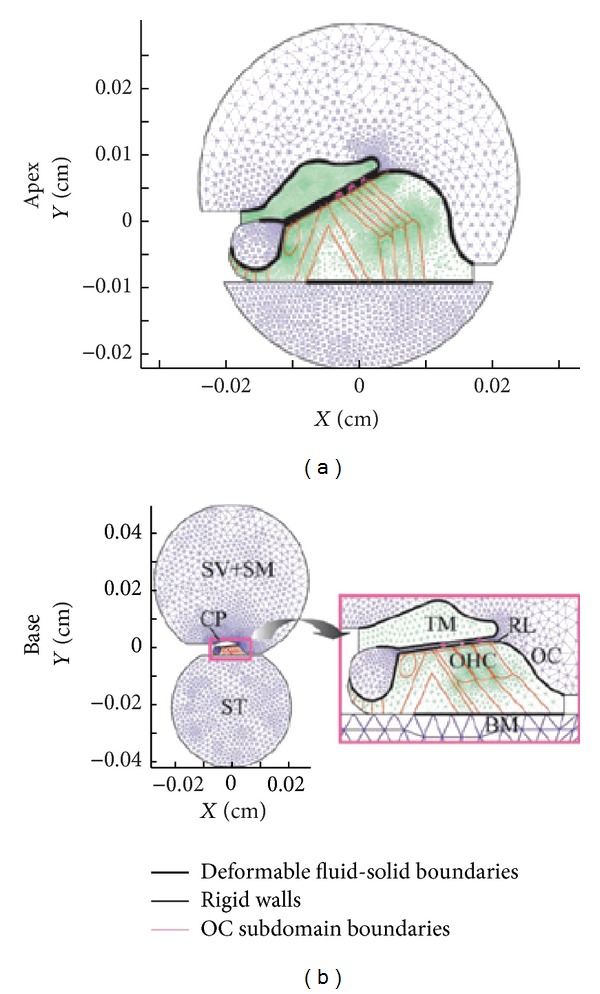
Geometry and mesh of cross-sections at apical (a) and basal (b) regions of the cochlea. *X* and *Y* indicate the radial and transverse directions, respectively. The TM and OC are modelled as 2D elastic domains. The TM is homogeneous, whereas the OC contains different subdomains representing discrete cellular structures. The OC has the RL as its top boundary and rests on the BM, which is represented by an orthotropic clamped plate. The TM-RL gap is the narrow fluid-filled space between the RL and the lower surface of the TM. Stereocilia of the OHCs elastically couple the RL and TM (reprinted from PNAS, 101, Cai et al., Evidence of Tectorial Membrane Radial Motion in a Propagating Mode of a Complex Cochlear Model, 6243–6248, Copyright (2004) National Academy of Sciences, USA).

Cai and Chadwick [90] developed a hybrid approach for modelling the apical end of guinea pig cochlea. In their FE cochlear model, they carry out only the first step in the reduction of the 3D hydroelastic problem to a sequence of eigenvalue problems in transverse planes. Then they used a WKB-numerical hybrid approach to do this reduction and provided the formalism for connecting the solution in different transverse planes via an energy transport equation. Later, they [91] used a similar approach to model cross-sections of the guinea pig cochlea at several positions, as shown in Figure 13, along the cochlea and solved the fluid-solid interaction eigenvalue problem for the axial wavenumber, fluid pressure, and vibratory relative motions of the cochlear partition as a function of frequency. Computations are done separately for each section which is believed to be the main computational advantage of their method, which relies on the WKB approximation. The fluid compartments are comprised of viscous, incompressible fluid with dynamics following the linearized Navier-Stokes equations. The solid domains (TM and OC) are modelled as linear isotropic Voigt solids with *E* replaced by a complex term to account for damping in the solid. The extracellular fluid spaces and tunnel spaces in the OC are not treated as fluid domains but are simplified to be soft Voigt solids. The BM is treated as an orthotropic plate, and the TM and RL are elastically coupled through the stereocilia bundle stiffness. The OHCs are treated as passive structural elements. Based on this 2D model, they retain coupling in the axial direction through the wavenumber *k* both in the fluid and solid domains.

**Figure 14:**
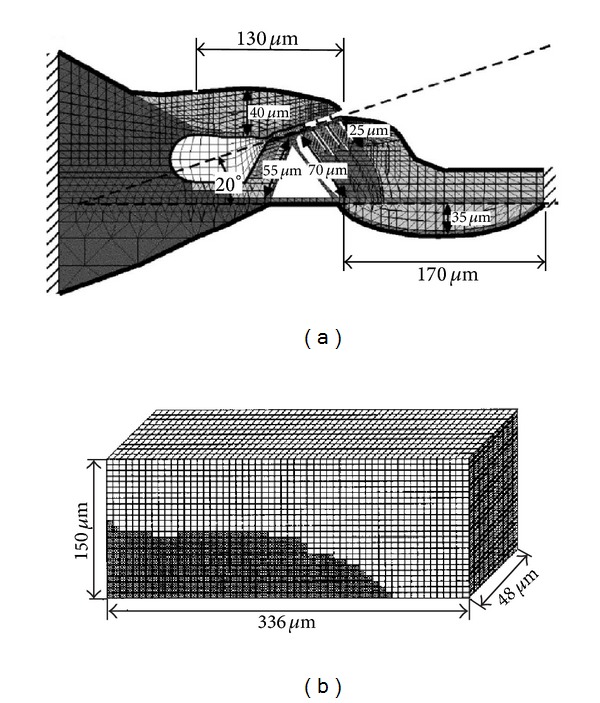
(a) 2D FE Model of the OC [93] (reprinted with permission from Journal of the Acoustical Society of America, 118, Andoh et al., Phase of Neural Excitation Relative to Basilar Membrane Motion in the Organ of Corti: Theoretical Considerations, 1554–1565, Copyright (2005), Acoustic Society of America) and (b) 3D scala vestibuli with rigid boundary conditions, in which dark area corresponds to the OC [92] (reprinted with permission from Journal of the Acoustical Society of America, 116, Andoh and Wada, Prediction of the Characteristics of Two Types of Pressure Waves in the Cochlea: Theoretical Considerations, 417–425, Copyright (2004), Acoustic Society of America).

Andoh and Wada used a finite element method to predict the characteristics of two types of cochlear pressure waves, fast and slow waves [92], and later estimated the phase of the neural excitation relative to the BM motion at the basal turn of the gerbil, including the fluid-structure interaction with the lymph fluid [93]. A two-dimensional finite element model of the OC, as shown in Figure 14(a), including fluid-structure interaction with the surrounding lymph fluid, was constructed based on measurement in the hemicochlea of the gerbil [94]. They assumed that the cross-section of the OC maintains its plane surface when external force was applied. Meshing was done at a subcellular level using a triangular element, by which the number of nodes and elements are 1,274 and 2,139, respectively. The fluid within the Corti tunnel was treated as an elastic body without shear stiffness. The viscous force was considered analytically on the assumption that Couette flow occurs in this space. The effect of the mass of the fluid in the subtectorial space was assumed to be negligible. The SV, as shown in Figure 14(b), and the ST were constructed in a 3D form to simulate the behaviour of the lymph fluid and its interaction with the OC. The dynamic behaviour of the local section of the OC, which extends in the longitudinal direction, was simulated and longitudinal widths of both fluid models were determined to be 48 *μ*m, which was less than one-fourth of the wavelength of the traveling wave [95]. A grid with intervals of 6 *μ*m was adapted to evaluate the pressure distribution around the OC in the scala. As a result, the SV model and the ST model had 11,200 and 8,000 cubic elements, respectively.

Kim et al. [96] developed a finite element model of a human middle ear and cochlea to study the mechanisms of bone conduction hearing. The geometry of the cochlear model was based on dimensions published in the literature [97] similar to the actual curved geometry of the cochlea. The BM was meshed with 14,000 8-node hexahedral solid shell elements, BM supports were meshed with 13,687 six-node pentahedral elements, and the RW was meshed with 1,719 six-node pentahedral elements. The nodes along the perimeter of the RW were fixed. The SV and ST were meshed with 222,350 4-node linear tetrahedral elements. The thickness of the bony shell, the rigid structure of the cochlea, was assumed to be 0.2 mm.

Finite element models have also been used to investigate the effects of several longitudinal coupling mechanisms on the coupled BM response [20, 24, 86, 91, 98]. Elliott et al. [57] used the wave finite element method to decompose the response of the fully coupled finite element model into the components due to each wave to study how they interact, which provides a way to give insight on numerical models that incorporate various detailed features of the cochlea, and allow the analysis of the contribution of each element in the OC to the overall response.

### 2.4. Waves in the Cochlea

Our understanding of the cochlea is largely based, either explicitly or implicitly, on the assumption that only a single type of wave propagates along its length. The properties of this “slow wave” can be calculated from a simple model of the passive cochlea that includes a locally reacting BM and 1D fluid coupling. In general, however, there are many other mechanisms, apart from 1D fluid coupling, that give rise to longitudinal coupling in the cochlea, particularly, the higher-order modes associated with 3D fluid coupling [57].

The discussion of multiple wave types in the cochlea is not new. Steele and Taber [23] and Taber and Steele [55], for example, used a Lagrangian approach to derive a dispersion relation, corresponding to the Eikonal equation in the WKB method, for waves in the passive cochlea. For 2D and 3D fluid coupling, the effective height of the fluid chamber is a transcendental function of the wavenumber and this leads to an infinite number of wavenumbers that satisfy the dispersion equation and hence multiple wave types. These authors note that the most difficult part of their numerical computation is the extraction of “the necessary root” of this equation that corresponds to a travelling wave solution that they are seeking. Their WKB solutions are then constructed from this single wave type. Similarly de Boer and Viergever [56] derived dispersion equations for 2D and 3D fluid coupling, noting that they have multiple roots and describe methods by which a single wavenumber may be selected corresponding to “the correct solution.”

These authors, and Steele and Taber [23], noted a difference between the WKB solution for the distribution of the complex BM motion along the cochlea and the full numerical solution, just apical of peak response. de Boer and Viergever [56] suggested that this is because the “wrong” solution of the dispersion equation has been chosen. Chadwick et al. [99] described an analytic model of a slice of the cochlea having subpartitions and four fluid chambers. They also derived a dispersion equation, which in their case is quartic and so yields four roots. It is noted that some roots represent nonpropagating waves and a single wavenumber was chosen for a given model to represent the propagating wave in their asymptotic formulation. Steele [100] also describes how multichamber models give rise to multiple modes. Cai and Chadwick [90] discussed how a more detailed numerical model of slices of the cochlea can be used to describe wave propagation. In this case a finite element model of the 2D cross-section was constructed and used to calculate multiple values of the wavenumber, from which the one with the least-negative imaginary part is selected for a WKB solution over the length of the cochlea. In each of these models, it has been assumed that a single wave type dominates the overall response of the cochlea. Watts [101] returned to the observed difference between the numerical and WKB solutions beyond the peak and discussed how a second wave mode could be introduced, which is necessary to satisfy the fluid coupling equation, that could explain this difference. There has also been recent interest in mode conversion in a two-chamber model of the cochlea [102].

Elliott et al. [57] used the wave finite element method [103], WFE, which was originally used to analyse wave propagation in uniform engineering structures such as railway lines [104] and tyres [105] to analyse a box model of the cochlea into its constitutive wave components. The WFE was used to calculate the position-dependent characteristics of the waves that are able to propagate through individual sections of a cochlear model. An advantage of this method over that described by Cai and Chadwick [90], for example, is that these sections can have a finite length and hence internal structure, although this aspect of the method is not exploited here. The main difference between this WFE model and other models, however, is that the calculated properties of these different wave types can be readily used to decompose the results of a full finite element analysis into individual wave components. They suggested that the response beyond the peak involves multiple wave types, however, as predicted by Watts [101], which are identified as higher-order acoustic waves in the fluid coupling. Following this, Ni and Elliott applied the WFE to predict wave propagations in an active, but still locally reacting, cochlear model. This active model uses the same elements as the passive one [57] but simulates the active impedance by using a complex and frequency-dependent Young's modulus in its finite element model of the BM. The BM velocity distributions and fluid chamber pressure distributions for the first few waves, which propagate with least attenuation, are similar in the active and passive cases due to the fact that the same finite element model is used for both, even though the material properties are different. The real part of the wavenumber for the slow wave has a higher peak value for the active model, indicating a smaller wavelength. The most significant difference, however, is that the imaginary part of the wavenumber for the slow wave is positive just before the peak position showing that the wave is amplified there. Although the properties of the slow wave are modified by the active components of the BM impedance, the other waves are still determined by the evanescent higher-order fluid modes.

It is only when additional forms of longitudinal coupling are included in the model, such as provided by multiple fluid chambers [99, 100, 102], that multiple propagating modes might be expected. There are, however, a number of other mechanisms for longitudinal coupling along the BM and it is unclear how these might behave together or interact with multiple fluid chambers, to determine the types of wave that can propagate. These mechanisms include orthotropy in the BM [106], tectorial membrane elasticity [107–109], longitudinal electrical coupling between the hair cells [21], and the feedforward action of the OHCs [12, 110].

## 3. Fluid Coupling

As described in Section 2.1.2 (elemental cochlear model), the linear coupled behaviour of the cochlear dynamics can be represented by two separate phenomena: the way that the pressure distribution is determined by the fluid coupling within the cochlear chambers when driven by the BM velocity and the way in which the BM dynamics respond to the imposed distribution of pressure difference.

**Figure 15:**
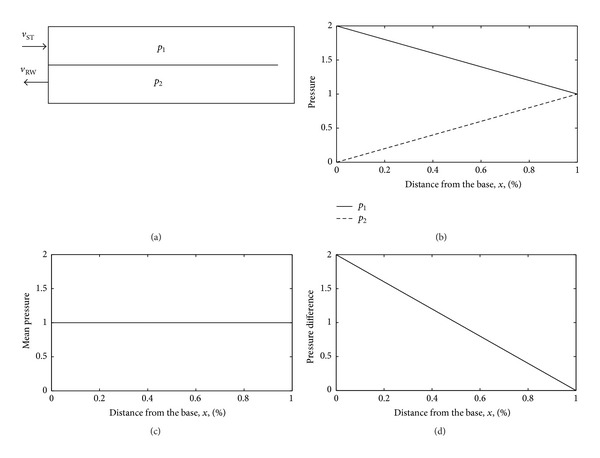
(a) The box model of the cochlea, (b) the pressure distributions in the upper and lower chambers as *p*_1_ and *p*_2_, (c) the mean pressure, and (d) the pressure difference.

When the box model of the cochlea with a rigid BM, Figure 15(a), is driven by the stapes, there are pressure distributions in the upper and lower chambers shown as *p*_1_ and *p*_2_ in Figure 15(b). These can be decomposed into a uniform mean pressure [111], $\overset{-}{p}=\left(p_{1}+p_{2}\right)/2$, in Figure 15(c), which gives rise to a fast wave that does not drive the BM and a pressure difference, $\overset{-}{p}=p_{1}-p_{2}$, which gives rise to a slow wave that does drive the BM.

### 3.1. Fluid Coupling in the Cochlea

**Figure 16:**
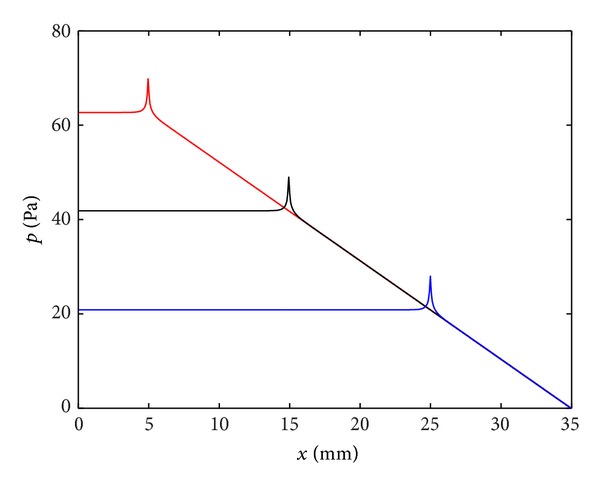
Distribution of the total pressure difference, due to both far and near-field components in the fluid coupling matrix, along the length of the cochlea due to excitation of a single element on the BM at *x* = 5 mm, 15 mm, or 25 mm with a velocity of 10 mms^−1^ at a frequency of 1 kHz.

The 1D fluid coupling assumed above is only valid when the height of the fluid chamber is small compared with the wavelength [12]. While this assumption is not unreasonable for the passive cochlear model, it breaks down as soon as an active model is being considered, since the wavelength of the slow wave in this case can be less than the size of the fluid chambers, particularly, at the base. More complete models of the fluid coupling must include the three-dimensional fluid effects that occur close to the BM, and the original formulation for 3D fluid coupling was presented in the wavenumber domain [23]. More recent formulations in the spatial and acoustic domains have been developed [70], which consider the fluid coupling to be the sum of the components due to far field, 1D, effects and to near-field effects, as illustrated in Figure 16.

Generally, a cochlear box model is a three-dimensional representation of the cochlea, since the fluid inside has the ability to move in all directions. Following Steele and Taber [23], in the wavenumber domain for the box model of the cochlea, the box is assumed to be symmetric; that is, the two fluid chambers above and below the BM are of equal area. The pressure distributions in the two chambers are thus equal and opposite and it is convenient to work with the single distribution *p*(*x*, *y*, *z*), equal to the pressure difference, which is twice the pressure in each chamber. The fluid is assumed to be incompressible and inviscid and so the conservation of fluid mass then leads to the equation

(21) $$\begin{array}{c} \frac{\partial^{2}p\left(x,y,z\right)}{\partial x^{2}}+\frac{\partial^{2}p\left(x,y,z\right)}{\partial y^{2}}+\frac{\partial^{2}p\left(x,y,z\right)}{\partial z^{2}}=0. \end{array}$$

The bony structures outside the cochlear fluids can be represented by hard boundary conditions on the sides and the top of the cochlear chamber above the BM, so that following relations must hold ∂*p*(*x*, *y*, *z*)/∂*y* = 0 at *y* = 0 and *y* = *W*, and ∂*p*(*x*, *y*, *z*)/∂*z* = 0 at *z* = *H*, where *W* and *H* are width and height of the fluid chamber. Since the BM separates the two fluid chambers, the fluid velocity at *z* = 0 must match that of the BM, so that ∂*p*(*x*, *y*, *z*)/∂*z* = −2*iωρv*_BM_(*x*, *y*) at *z* = 0, where the factor of 2 is due to the pressure doubling when *p*(*x*, *y*, *z*) is defined as the pressure difference.

The BM velocity is now assumed to have a given distribution across its width, and in the longitudinal direction it has a sinusoidal variation with wavenumber *k*, so that

(22) $$\begin{array}{c} v_{\text{BM}}\left(x,y\right)=v\left(x\right)\psi \left(y\right)=V\left(k\right)\psi \left(y\right)e^{-ikx}, \end{array}$$

where *v*(*x*) is the “modal” BM velocity distribution along the cochlea and *ψ*(*y*) is the BM velocity distribution in the transverse direction.

The distribution of the transverse motion across the width of the BM is complicated and level-dependant in the real cochlea [53, 112]. Homer et al. [113] developed a beam model of the BM to study the effect of boundary conditions at the two ends and compared their predictions with experimental data [112]. They found that the best fit is obtained by assuming the BM is simply supported at the arcuate end and clamped at the other end. Steele et al. [114] used a similar beam model, which is simply supported at the arcuate end and clamped at the other end, but with an attached spring to simulate the outer pillar, to compare the radial profile of displacement of the BM with that from experiment [112]. They compared the cases with both a pressure load and a point load and found that by setting the effective spring constant to zero, the model has a good fit with the profile of displacement with the pressure loading. Ni and Elliott [78] investigate the effects of BM radial velocity profile, *ψ*(*y*) on the fluid coupling in the cochlea. Although experimental observations [112] and modelling studies [113] suggest that the best fit to experimental data is the BM mode shape obtained when the BM is simply supported at the arcuate end and clamped at the other end, they find that the fluid coupling and the coupled response are not critically dependent on the tested boundary conditions for the BM.

The normalised BM velocity distribution, *ψ*(*y*), in the box model of the cochlea, as shown in Figure 8, can be given by

(23) $$\begin{array}{c} \overset{W}{\underset{0}{\int }}\psi^{2}\left(y\right)dy=W, \end{array}$$

so that *v*(*x*) can be calculated from *v*_BM_(*x*, *y*) as

(24) $$\begin{array}{c} v\left(x\right)=\frac{1}{W}\overset{W}{\underset{0}{\int }}v_{\text{BM}}\left(x,y\right)\psi \left(y\right)dy. \end{array}$$

The pressure field can be described by a summation of modes of the form

(25) $$\begin{array}{c} p\left(x,y,z\right)=\overset{\infty }{\underset{n=0}{\sum }}B_{n}\phi_{n}\left(y,z\right)e^{-ikx}, \end{array}$$

where each mode shape, *ϕ*_*n*_(*y*, *z*), must satisfy the boundary conditions defined. A suitable parameterisation of the pressure mode shape [23, 77] is

(26) $$\begin{array}{c} \phi_{n}\left(y,z\right)=\cos\left(\frac{n\pi y}{W}\right)\cosh\left[m_{n}\left(z-H\right)\right]. \end{array}$$

In order for each term in the model expansion to satisfy the equation for mass conservation, (21), then the real parameter *m*_*n*_ must satisfy the equation

(27) $$\begin{array}{c} m_{n}^{2}=k^{2}+\frac{n^{2}\pi^{2}}{W^{2}}. \end{array}$$

The coefficients *B*_*n*_ are determined by the boundary condition at the BM, so that

(28) ∑n=0∞Bn∂ϕn(y,z)∂z  =−2iωρV(k)ψ(y), at  z=0.

Substituting (26) into (28) gives

(29) ∑n=0∞Bnmnsinh⁡⁡(mnH)cos⁡⁡(nπyW)  =2iωρψ(y)V(k).

Multiplying each side of (29) by cos⁡⁡(*nπy*/*W*) and integrating from 0 to *W* over *y* and using the orthogonality of the cos⁡⁡(*nπy*/*W*) function yield

(30) $$\begin{array}{c} B_{n}=\frac{2i\omega \rho A_{n}}{m_{n}\sinh\left(m_{n}H\right)}V\left(k\right), \end{array}$$

where the coupling coefficient for *n* = 0 is defined as

(31) $$\begin{array}{c} A_{0}=\frac{1}{W}\overset{W}{\underset{0}{\int }}\psi \left(y\right)dy, \end{array}$$

and for *n* ≥ 1 is

(32) $$\begin{array}{c} A_{n}=\frac{2}{W}\overset{W}{\underset{0}{\int }}\cos\left(\frac{n\pi y}{W}\right)\psi \left(y\right)dy. \end{array}$$

The modal pressure can be written by analogy with the modal velocity in (22) as [70]

(33) $$\begin{array}{c} p\left(x\right)=P\left(k\right)e^{-ikx}, \end{array}$$

where

(34) P(k)=2iωρ[A02kcoth⁡⁡(kH)   +∑n=1∞An22mncoth⁡⁡(mnH)]V(k).

In the wavenumber domain, the pressure difference can be represented by [70]

(35) $$\begin{array}{c} P\left(k\right)=2i\omega \rho Q\left(k\right)V\left(k\right), \end{array}$$

where *Q*(*k*) has the dimensions of length and has been termed the “equivalent height” [115]. For the 3D case, *Q*_3D_(*k*) is given by

(36) Q3D(k)=A02kcoth⁡⁡(kH)+∑n=1∞An22mncoth⁡⁡(mnH).

Based on the 3D expression of the fluid coupling in the cochlear, 1D and 2D expressions can be obtained by some simplifications. For example, the fluid component can be simplified to a one-dimensional function of only longitudinal position. In two-dimensional models, the height of the fluid is taken into account and in the three-dimensional models the width of the fluid and the width of the cochlear partition are additionally included. For the two-dimensional model, the pressure associated with the first term in (36) corresponds to the pressure* zero* mode shape and has no radial variation [71], and the equivalent height for this case can be written as

(37) $$\begin{array}{c} Q_{2\text{D}}\left(k\right)=\frac{8B}{\pi^{2}Wk}\coth\left(kH\right). \end{array}$$

Using the long-wavelength approximation with the one-dimensional model, in which the wavelength is large compared to *H*, so that *kH* is significantly less than unity, the equivalent height for the one-dimensional fluid model can be given by

(38) $$\begin{array}{c} Q_{1\text{D}}\left(k\right)=\frac{8B}{\pi^{2}WHk^{2}}. \end{array}$$

**Figure 17:**
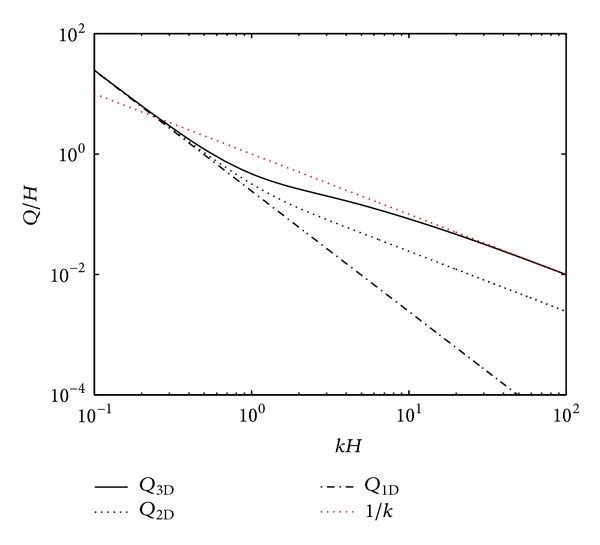
The normalised fluid equivalent height *Q*(*k*)/*H* as a function of normalised wavenumber, *kH*. In this example, the BM is assumed to be located at the edge of the CP and the width of the BM is one-third of the CP. The assumed boundary conditions for the BM are simply supported at the arcuate end and clamped at the other end.

For low values of *kH*, the wavelength of the longitudinal BM vibration is much greater than the height of the fluid chamber, and so 1D fluid coupling, *Q*_1D_, is nearly identical to 2D and 3D fluid coupling, *Q*_2D_ and *Q*_3D_, as shown in Figure 17, and thus the pressure is almost uniform across the cross-sectional area. As the wavelength becomes comparable with the height, the difference among different models becomes significant. When the wavelength is small compared with the height, *Q*_3D_ becomes proportional to 1/*k*, which is larger compared with *Q*_2D_ and *Q*_3D_, as shown in Figure 17, and the pressure is much greater closer to the BM than it is in the rest of the fluid chamber. Thus when the wavelength is small compared with the height of the fluid chamber, that is, near CF, 1D and 2D models do not well represent the cochlear mechanics, since they do not have ability to take the increase of the local mass loading [116] caused by BM resonance into account.

### 3.2. Modal Description of the Fluid Coupling

The Green's function was widely used for calculating the fluid coupling, for example, by Allen [117], Mammano and Nobili [31], and Shera et al. [118]. This method is, however, having singularity in the near-field component due to the fact that the vibrating element is a spatial delta function [20, 31, 119]. This singularity can be avoided if the imposed BM velocity is assumed to act over a finite length, as given by (19) in Elliott et al. [70]. Alternatively, the distribution of the fluid pressure can also be described as a sum of different modes analogous to an analysis of the acoustic field due to an elemental source in a duct as described by Doak [120]. The complex pressure, for positive values of *x*, due to a point monopole source of volume velocity *q*_0_, at location *x* = 0, *y* = *y*′, and *z* = *z*′ within a single cochlear chamber, modelled as a hard walled rectangular duct, can be expressed as

(39) $$\begin{array}{c} p_{c}\left(x,y,z\right)=\overset{\infty }{\underset{m=0}{\sum }}B_{m}\phi_{m}\left(y,z\right)e^{-ik_{m}x}. \end{array}$$

Only forward travelling waves are assumed, *m* denotes a duo of modal integers, *m*_1_ and *m*_2_, *k*_*m*_ is the modal wavenumber, and *ϕ*_*m*_(*y*, *z*) represents the assumed acoustic mode shape

(40) $$\begin{array}{c} \phi_{m}\left(y,z\right)=\sqrt{\varepsilon_{m_{1}}\varepsilon_{m_{2}}}\cos\left(\frac{m_{1}\pi y}{W}\right)\cos\left(\frac{m_{2}\pi z}{H}\right). \end{array}$$

The normalization constants *ε*_*m*_1__ and *ε*_*m*_2__ are equal to 1 if *m*_1_ or *m*_2_ equal zero and are otherwise equal to 2, so that the mode shapes are orthonormal, such that

(41) ∫y=0W∫z=0Hϕn(y,z)ϕm(y,z)dy dz=WH,m=n,∫y=0W∫z=0Hϕn(y,z)ϕm(y,z)dy dz=0,m≠n.

The modal amplitude in (39) is given by

(42) $$\begin{array}{c} B_{m}=\frac{\omega \rho q_{0}}{2Ak_{m}}\phi_{m}\left(y,z\right), \end{array}$$

where *A* is the cross-sectional area of the chamber, which is *WH* in this case.

The difference between this formulation and that in the wavenumber domain is that the driving source is initially assumed to be concentrated at a point, rather than the infinite sinusoidal distribution along the cochlea assumed in the wavenumber analysis, and that instead of the wavenumber being a specified value, it is now a variable that changes with the modal order. In the case assumed here, where the fluid is assumed to be incompressible, the modal wavenumber becomes

(43) $$\begin{array}{c} k_{m}=\pm i\sqrt{{\left(\frac{m_{1}\pi }{W}\right)}^{2}+{\left(\frac{m_{2}\pi }{H}\right)}^{2}}, \end{array}$$

which can be written as ±*i*/*l*_*m*_. Provided *m*_1_ and *m*_2_ are not both zero, corresponding to a fast wave of infinite speed, the modal contributions are thus all evanescent, with a longitudinal dependence that can be written, by choosing the appropriate root of *k*_*m*_, as

(44) $$\begin{array}{c} e^{-ik_{m}x}=e^{-x/l_{m}}, \end{array}$$

where *l*_*m*_ is a modal decay length.

The pressure in the chamber due to the velocity distribution corresponding to excitation of a single element of the BM with a predefined modal shape *ψ*(*y*) can also be calculated from (39), by generalizing (42) to give the modal amplitude for a distribution of monopole sources [120], so that the modal amplitude can be obtained by integrating over the area of the element:

(45) Bm=ωρq0WHkm∫0Wψ(y)ϕm(y,0)dy(∫−Δ/20ex/lmdx+∫0Δ/2e−x/lmdx).

The modal pressure difference due to the far field component is thus due to the plane acoustic wave, corresponding to both *m*_1_ and *m*_2_ equal to zero. The near-field component of the modal pressure can then be calculated, for *m* greater than zero, by integrating the pressure in (39) over the BM width, to give

(46) $$\begin{array}{c} P_{N}\left(x\right)=\frac{2}{W}\overset{W}{\underset{0}{\int }}\psi \left(y\right)p\left(x,y,0\right)dy. \end{array}$$

The modal pressure due to the near-field of this vibrating element of the BM can thus be written as

(47) $$\begin{array}{c} p_{N}\left(x\right)=\overset{\infty }{\underset{m=1}{\sum }}a_{m}e^{-x/l_{m}}, \end{array}$$

where *a*_*m*_ is the overall modal amplitude. Each mode has its own decay length *l*_*m*_, and it is clear from (43) and the definition of *l*_*m*_ that these become increasingly small as *m* becomes larger, resulting in a more local response, which is enhanced by the fall off in the mode amplitude, *a*_*m*_, with *m*. The lowest order evanescent mode, for which *m*_1_ = 0 and *m*_2_ = 1, has a decay length, *l*_*m*_, which is equal to *H*/*π*. The condition under which the effect of the near-field pressure can be lumped together as a local mass [77] is thus that *H*/*π* is small compared with the wavelength of the cochlear wave. This is a somewhat more restrictive condition than the conventional, long wave, assumption for 1D fluid coupling, which is that 2*πH* should be less than the wavelength [12].

**Figure 18:**
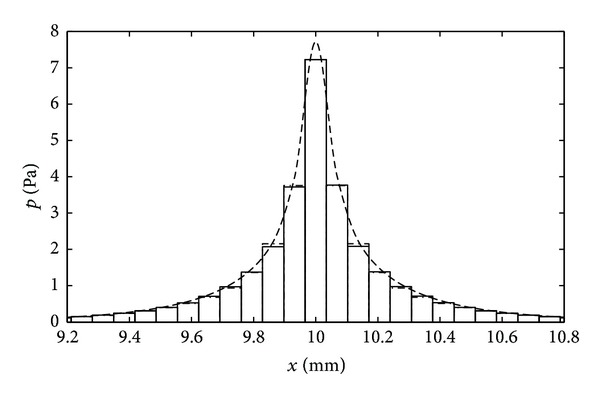
Continuous distribution of the modal pressure along the cochlea due to the fluid coupling near-field component (dashed line) and the average pressure over each discrete element of the BM (solid line), when excited by a single element at *x* = 10 mm with a velocity of 10 mm s^−1^ at a frequency of 1 kHz. Also shown (dot-dashed line) is the approximation to this discrete distribution obtained from the sum of two exponentially decaying terms of an acoustic analysis of the fluid coupling, (48).

In fact, a reasonable approximation to the averaged near-field pressure due to a single BM element can be obtained using only two terms of the infinite series in (47), so that in the discrete model [121]

(48) $$\begin{array}{c} p_{NA}\left(n^{\prime }\right)=2i\omega \rho \left(Q_{1}e^{-n^{\prime }\Delta /l_{1}}+Q_{2}e^{-n^{\prime }\Delta /l_{2}}\right)v_{0}, \end{array}$$

where *n*′ is equal to |*n* − *n*_0_| for excitation of the *n*_0_th element, *Z*_1_ and *Z*_2_ are two impedances, and *l*_1_ and *l*_2_ are the corresponding characteristic decay lengths. This approximation to the average pressure over the discrete elements is also shown in Figure 18, with equivalent height *Q*_1_ and *Q*_2_ equal to 16 *μ*m and 41.56 *μ*m, *l*_1_ equal to *H*/3.47, and *l*_2_ equal to *H*/12.8, and is seen to provide a good approximation to the result obtained from the inverse Fourier transform of (35).

### 3.3. Finite Element Modelling of the Fluid Coupling

The finite element method is a powerful technique that has the advantage of modelling complex structures. In the finite element model, the fluid coupling (of the box model or of a complex geometry such as a coiled model) of the cochlea can be written as

(49) $$\begin{array}{c} Q{\ddot{p}}_{\text{FE}}+Hp_{\text{FE}}=q_{\text{FE}}, \end{array}$$

where **Q** is the mass matrix, **H** is the stiffness matrix, **q**_FE_ is the BM velocity vector, and **p**_FE_ is the vector of pressures at all of the nodes [76]. Consistent with the fluid coupling models mentioned above, the imposed velocity at the BM should have a predefined radial profile.

**Figure 19:**
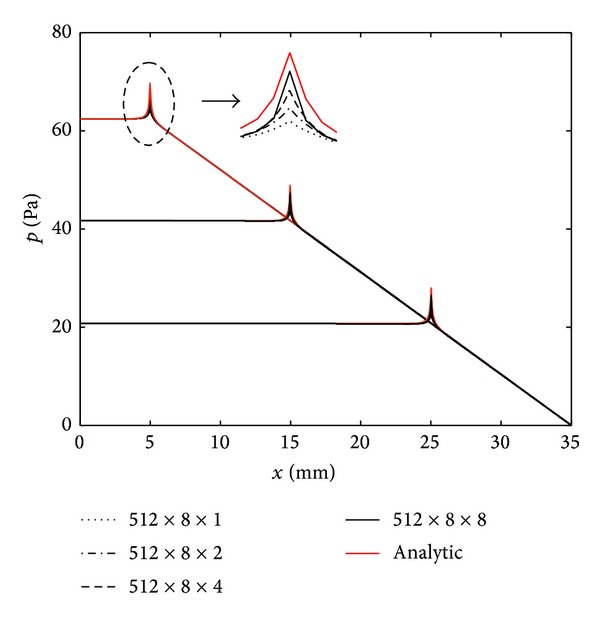
Modal pressure difference on the BM calculated using the FE model for excitation of a single longitudinal segment of the BM at *x* equal to 5 mm, 15 mm, and 25 mm with a velocity of 10 mm*·*s^−1^ at a frequency of 1 kHz with 8 × 1 elements (dotted lines), 8 × 2 elements (dashed lines), 8 × 4 elements (dot-dashed lines), 8 × 8 elements (solid lines), and analytic solution (red lines) [121].

The rectangular box geometry needs to be divided into finite longitudinal sections to fulfil the requirement that there are at least 6 elements within the shortest wavelength, which is a common rule in finite element analysis [122]. The meshing in the cross-section has to be finer than this in order to capture the near-field pressure variation close to the vibrating BM [61].  Figure 19 shows the distribution along the cochlea of the computed modal pressure difference on the BM, when driven by a single longitudinal BM segment at different locations, for various mesh sizes in the FE model [121]. It can be seen that with relatively few elements, the FE model reproduces the long wavelength, far field, behaviour of the pressure reasonably well, but a larger number of elements are required to reproduce the near-field pressure on the BM and hence the additional short wavelength component of the modal pressure. The results with the smaller mesh size are in good agreement with those computed from the analytic models [70].

An advantage of the finite element method is that since the fluid is modelled using acoustic elements, the compressibility of the fluid, as well as its inertial properties, is taken into account. The widely used theoretical models [23, 56, 123] assume that the fluid is incompressible. The effects of compressibility are expected to be greater at higher frequencies as the inertial forces become larger. In the incompressible model, the fluid pressure would be independent of frequency. However, the magnitude and shape of the fluid pressure changes significantly with frequency in the finite element model [124]. The magnitude increases at a quarter wavelength resonance, which is about 10 kHz for the human cochlea with a length of 35 mm, and the distribution of fluid pressure is no longer linear away from the excitation point. This acoustic resonance increases the magnitude of the average pressure across any cross-section of the cochlea, but does not influence the short wavelength components which are unaffected by the compressibility of the fluid [125]. The resonant peak at the frequency of a quarter wavelength resonance is accompanied by a phase change, so that the pressure distributions for excitation frequencies above and below that frequency are almost entirely out of phase.

It is interesting to compare the predicted frequency of this quarter wavelength resonance with the upper frequency of hearing in several species [126, 127]. This resonance appears to occur, perhaps coincidentally, at about half the upper frequency limit of hearing in each of these cases. Although this acoustic resonance is, in retrospect, simple to predict, its existence for the pressure difference component and its effect on cochlea mechanics does not appear to have previously been widely considered. Peterson and Bogert [111] and Lighthill [125] discuss a quarter wavelength resonance in the mean pressure component, but this is associated with a pressure source driving the cochlea as a closed duct, in order to match the pressure release boundary condition at the round window.

If the cochlear fluid is assumed to be compressible, then the classical slow cochlear wave can be given by [111, 124, 128]

(50) $$\begin{array}{c} k\left(x,\omega \right)=\sqrt{\frac{-2i\omega \rho }{h}{\text{Y}}_{\text{BM}}\left(x,\omega \right)+\frac{\omega^{2}}{c_{0}^{2}}}, \end{array}$$

where *c*_0_ is the speed of sound in the cochlear fluid. The wavenumber is thus not significantly affected by the compressibility, since the maximum speed of the slow wave is about 70 m/s, which is much smaller than the 1,500 m/s speed of the fast wave.

Despite the very significant change in the pressure distributions in the fluid coupling calculations due to fluid compressibility, this hardly appears to have any effect on the coupled cochlea response at all. This surprising result can perhaps be understood by returning to the way in which the coupled model is formulated [70]. The fluid coupling effects are first calculated independently of any BM motion by defining the fluid coupling impedance matrix, for the fluid chambers having rigid walls. It is this assumption that leads to the quarter wavelength resonance in the uncoupled fluid column. When the BM is allowed to move, in the coupled response, however, this resonance does not get a chance to become established, since the BM is sufficiently mobile that it substantially equalizes the pressures in the two fluid chambers well before the wave reaches the end of the cochlea.

### 3.4. Geometric Effect on the Fluid Coupling

**Figure 20:**
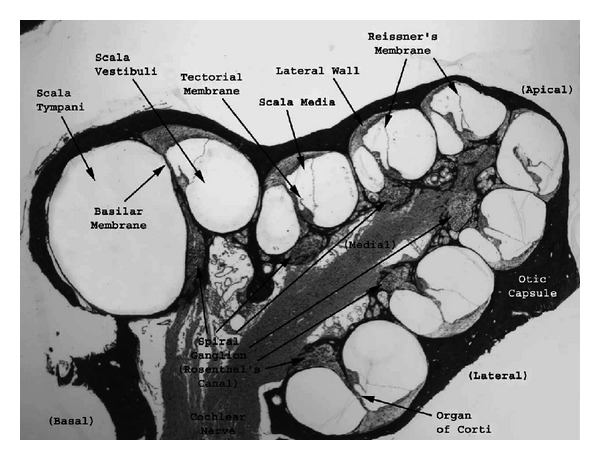
A micrograph of a plastic cross-section of the guinea pig cochlea. The section is cut at a near midmodiolar plane [142] (reprinted from Brain Research Bulletin, 60, Raphael and Altschuler, Structure and Innervation of the Cochlea, 147–154, Copyright (2003), with permission from Elsevier).

Due to the fact that the cochlear components of interests are housed in bone, as shown in Figure 20, it is difficult to describe them in an experimental way. Most cochlear mechanics researchers reduce the real cochlea structure into a simple mathematical model with assumed physical and geometrical properties [12, 23]. Elliott et al. [70] developed an elemental model of the cochlea in order to analyse the interaction between the fluid coupling and BM motion and represented the cochlear mechanics by defining a single longitudinal variable for the pressure difference and for the BM velocity. The effect of asymmetry of the cochlear structure has been discussed by assuming that the width of the BM varies along the cochlear length. They found that due to the reduction of the equivalent height with distance, the fall-off of the pressure difference beyond the driving position on the BM is no longer linear but has curvature, which was also observed by Shera et al. [118] using a Green's function approach. This indicates that the nonuniformity has a great effect on the changes in the wavelength of the BM motion as it approaches the characteristic place, since the effective area of the cochlear chambers becomes much less than that at the base. There is then a reduction in longitudinal fluid flow due to reflection and an increase in the local mass loading, slowing the wave and increasing the phase accumulation. However, other geometries, like the height of the fluid chamber and the width of the CP, were assumed to be constant. As an extension to Elliott et al. [70], Ni [121] developed a more general expression, which takes variations of the BM width, CP width, and fluid chamber height into account, to study the geometric effects on the fluid coupling.

Another important geometric factor in cochlear mechanics is coiling. It is believed that the coiled structure is an adaptation to the problem of fitting a long basilar membrane, to provide good low frequency hearing, into the relatively small heads of early mammals [129]. The origins of the coiled cochlea have recently been traced back 150 million years [130]. In morphogenesis of extant marsupials and placentals, the full coiling of the cochlear duct is inextricably linked with the formation of the cochlear ganglion and complex bony labyrinth structures, all during the late embryogenesis. Obviously, the coiled cochlea is a key evolutionary innovation of modern mammals. Despite providing a good blood and nerve supply, however, the effects of the coiling on the mechanics of the cochlea are still not fully understood.

von Békésy [4] states that the coiling is not essential as far as mechanics are concerned because a few animals, for example, the anteater, have a cochlea on the form of a slightly bent tube. The first mathematical attempt to analyse the possible mechanical effects of the spiral coiling was due to Huxley [131], who derived an ordinary differential equation for the pressure in an uncoiled 1D cochlear model similar to the box model which is widely used now and gave estimates indicating that coiling of the cochlear geometry could mechanically isolate adjacent sections along the cochlear partition and provide a sharp resonance effect. Hereafter, only a few researchers considered how spiral coiling may affect the BM dynamics, fluid coupling, and low frequency perception. Fleischer et al. [132] used a finite element model to study the effect of coiling on the stiffness distribution of the BM along the cochlea. They found that the coiling exerts its greatest influence on the apical third of the BM, although a much larger influence on the range of BM stiffness was the longitudinal variation of its thickness. This reinforced the earlier work of Viergever [66], who also concluded that the mechanical behaviour of the cochlea is only slightly affected by its spiral form.

An analytic model of the fluid coupling in the coiled cochlea was developed by Steele and Zais [133], who concluded that the response was not significantly affected by the coiling. Kohllöffel [134] also suggested that the effect of the coiling on the pressure difference is small and that there is an equivalent straight cochlea in the limit of long wavelength. The author also noted that the frequency of the quarter wavelength resonance in the mean component of the pressure is raised by about half an octave due to coiling. Manoussaki and Chadwick [135] considered fluid loading using an analytic model of the coiled “helical box” model of the cochlea using a wavenumber analysis and found that the fluid loading at the apex was only about 11% less in the coiled cochlea compared with the straight cochlea. In subsequent publications, however, Cai and Chadwick [90], Cai et al. [67], and Manoussaki et al. [136] emphasised the redistribution of wave energy towards the outer wall of the cochlea generating a radial force on the OC that significantly increases its shear gain at the apex, which can lower the fluid impedance at the apex, and thus helps detection of low frequency sounds. Fleischer et al. [132] developed a three-dimensional finite element model of the basilar membrane to explore the impacts of coiling and other factors such as material properties on the compliance of the unloaded basilar membrane. They find that the coiling has a weak influence on the BM compliance and the largest effect is in the apical third of the cochlea, where the curvature is the greatest. The increase of the BM stiffness is less than a factor of 1.6. They also suggested that the reduction of curvature in an isolated BM cannot achieve the 20 dB amplification found by Manoussaki et al. [136], which indicates that the fluid-structural coupling has a great effect on coiling, especially in the apical cochlear region.

Ni et al. [79] calculated the pressure difference between two fluid chambers using a three-dimensional finite element model of the coiled cochlea and found that the effects of the coiling on the far-field components are more obvious than that on the near-field components. The magnitude of the pressure difference has a reduction apical to the BM driving position compared with that from a nonuniform straight model. This implies that the fluid impedance decreases at those positions, which are close to the apex due to the spiral coiling of the cochlea being greatest at the apex. Following this, Ni [121] applied the elemental method [70] to study the effects of coiling on the coupled response by assuming that the BM dynamic is not affected by the coiling and the results show that the difference between the coiled and the straight model becomes larger at low frequencies, when the characteristic place moves towards the apex, reaffirming that the curvature plays a more important role close to the apical end of the cochlea.

## 4. Cochlear Micromechanics

The representation of the cochlea can be discussed in terms of its micromechanics and its macromechanics. The term “micromechanics” refers to the dynamic behaviour of a radial slice of the cochlea at the microscopic level. By contrast, the term “macromechanics” deals with the coupling between the micromechanical motion of the system at various points along the cochlea, thus giving rise to a solution for the global response of the cochlea.

Generally, all models used to describe the BM vibration patterns or the pressure distributions along the cochlea are dealing with macromechanics, since these models concern the interaction between fluids in the two fluid chambers with the CP (all the parts of which are assumed to move in a same manner with the BM). The longitudinal coupling of the CP, for example, the phalangeal processes which longitudinally connect OHCs, is neglected. The components within the OC can move with different magnitude and phase. Nowotny and Gummer [137] observed that under electrical stimulation the TM at both inner and outer radial positions vibrates in phase with the RL of the OHC, which in turn vibrates 180° out of phase with the RL of the IHC. This counterphasic motion of TM and RL at the IHCs is considered as the reason of pulsating fluid motion in the subtectorial space.

### 4.1. Passive Cochlear Micromechanics

**Figure 21:**
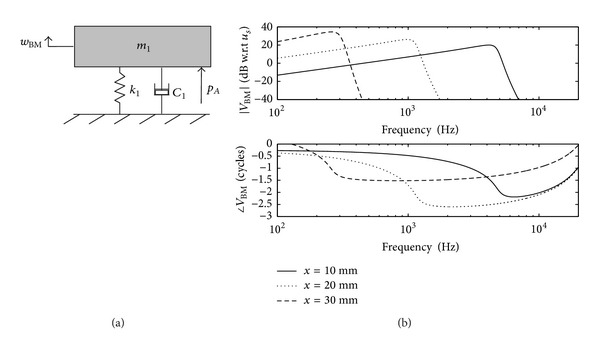
(a) One-degree-of-freedom micromechanical model, in which the BM and TM are assumed to always move parallel to each other without radial motion. (b) Distribution of the magnitude and phase of the BM velocity of the passive cochlea in frequency domain calculated using one-degree-of-freedom micromechanical model.

In the classic travelling wave model, the cochlea is taken as a hydromechanical element, determined by the physical structure of the cochlea, which provides the basis for frequency analysis. This passive, travelling wave model was first proposed by von Békésy [4], who measured the travelling wave in cadaver ears, using an optical method that required very high input levels to make the responses large enough to be observed. For this kind of behaviour, the response is not dependent on stimulus level, except for amplitude scaling, and is described as “passive.” Passive models of the cochlea, as reviewed by de Boer [12], for example, can provide predictions of the distribution of motion along the cochlea at a given frequency or of its frequency response at a given position. These models include the macromechanical behaviour of the fluid coupling along the length of the cochlea, as well as the micromechanical behaviour of the individual parts of the cochlear partition. Such models are the starting point for more realistic nonlinear models. In order to produce numerical results, the cochlear partition, which has mechanical parameters that vary continuously along its length, is often approximated by a discrete set of elements. This allows a finite dimensional set of equations to be solved one frequency at a time. The number of elements is generally quite large, typically about 500 for the human cochlear model, so that a single model, as shown in Figure 21(a), generates many hundreds of individual frequency response functions, as shown in Figure 21(b).

Comparing with the cochlea* in vivo*, the passive cochlea loses the active and nonlinear behaviours. The passive model is a reasonable representation of the actual cochlear response at high stimulus levels, above 80 dB SPL, since the cochlear amplifier is saturated and plays no role in its dynamics.

### 4.2. Active Cochlear Micromechanics

The direct physiological evidence of the active feedback process in the cochlea is the observation of sound in the ear canal caused by spontaneous oscillations, apparently of cochlear origin, retransmitted by the middle ear, which are called spontaneous otoacoustic emissions [10]. The basic mechanism of the active process in the* in vivo* cochlea can be explained as an interaction between the BM and the OHCs. When the BM moves upwards, the stereocilia of the OHCs are deflected by the shearing motion between the RL and the TM, which opens transduction channels and causes a change in the OHCs intracellular potential and thus a change in the length of the cell, which will generate a force upward acting on the BM from the OHCs through the Deiters' cells to enhance BM motion. So the micromechanics of the OC have to be considered as a closed-loop feedback system [75]. The idea of modelling the active function in the cochlea was developed in the 1970s and early 1980s and comprises two basic aspects: (1) the normal cochlear function depends on an active, mechanical feedback processes, and (2) OHCs operate as the agent of feedback. Generally, cochlear models that take OHCs motility into account are described as active models and those without OHC motility are defined as passive cochlear models [77]. Based on this, OHCs motility has been introduced into a number of active cochlear models to describe cochlear active amplification [32, 35, 80, 138–141].

#### 4.2.1. Modelling OHC Motility

**Figure 22:**
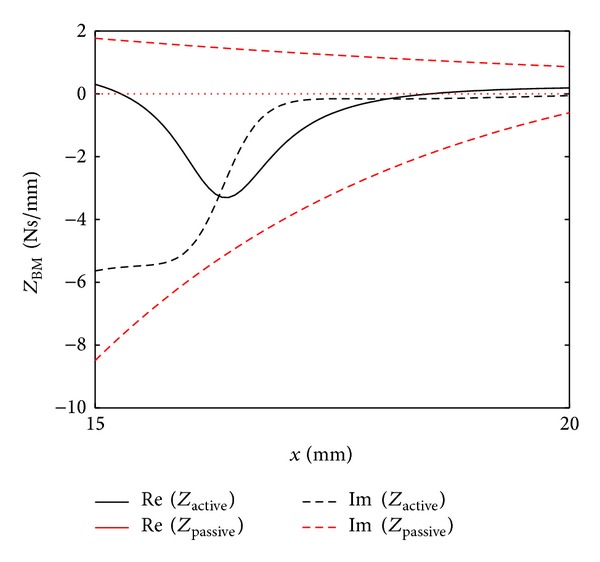
The distribution of the BM impedance at frequency of 1 kHz.

In the early stages of cochlear modelling, the models were formulated mechanically in the frequency domain and active undamping was assumed at a site basal to the characteristic place with only a single degree of freedom to represent the dynamics of the CP. This fixed the spatial distribution of undamping in the model, and thus the pattern of impedances was only valid for one frequency [143]. One way to represent the active behavior of the OHCs is the inclusion of negative damping, providing energy rather than dissipating. The first active cochlear model was proposed by Kim et al. [37]. The model is two-dimensional and has negative damping over a limited region. de Boer suggested [144] that it was impossible for a passive mechanical short wave model to have a “sharp response,” similar to that found in hair cell potentials or auditory nerve fibers. In his 1D lumped-parameter model, he defined the real part of the BM impedance, Z_BM_ = *iωm* + *s*/*iω* + *r*, to be negative in the region to the left of the resonance peak, as shown in Figure 22, to make the BM active in that region, which means the damping term, *r*, must be negative close to the peak response region. Diependaal et al. [39] developed a similar 1D model including active and nonlinear mechanisms and solved the model in the time domain using a fourth-order Runge-Kutta method.

Alternatively, Zweig [145] adopted negative damping to describe the active behavior in the OC based on a passive transmission-line cochlear model, in which OHCs were conjectured to be active elements contributing to the negative damping and feedback of the cochlear amplifier. Hubbard and Mountain [143] applied the second filter using a travelling wave amplifier in their active cochlear model. Following the finding of nonlinear BM activity and the existence of OAEs coupled with measurements of BM motion* in vivo* [5, 146–148], researchers began to incorporate active elements into their models. Comparing with 1 DOF models, a second degree of freedom is added to represent the TM above the BM by many researchers [19, 22, 28, 40, 107]. This allowed the active response to be generalized over the entire range of locations along the CP and thus the entire spectrum of audible frequencies [149].

**Figure 23:**
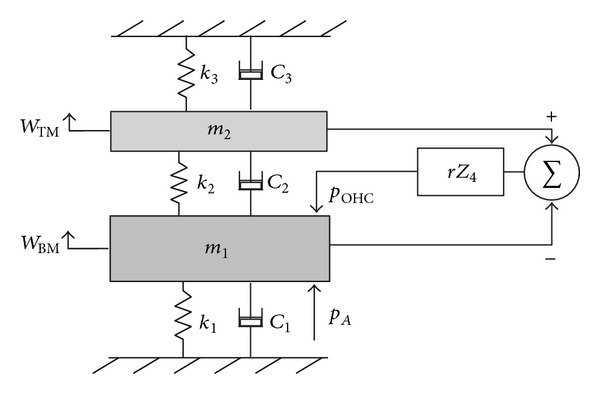
Mechanically equivalent system for Neely and Kim's 1986 model [19], in which *m*_1_ and *k*_1_ represent the transverse mass and stiffness of the BM and organ of Corti, and *m*_2_ and *k*_2_ represent the transformed effect of the radial motion of the tectorial membrane, TM. The model thus has two degrees of freedom. The active pressure due to the outer hair cells, *P*_A_, is assumed to be proportional to the difference in the displacement and velocity of *m*_1_ and *m*_2_ via the impedance, *Z*_4_, and cochlear amplifier gain, *r*.

A number of authors have extended the mass-spring-damper representation of the passive BM mechanics, to include lumped-parameter representations of the dynamics of the OC. These micromechanical models can then include forces due to the action of the outer hair cells in an attempt to represent the cochlear amplifier. A good review of such models is provided by Patuzzi et al. [150], although the most famous model was put forward by Neely and Kim [19] to represent the active cochlea in the cat. The equivalent mechanical system for Neely and Kim's 1986 model is shown in Figure 23. By careful selection of the values of the 10 mechanical parameters in this model, and their distribution along the cochlea, reasonable predictions of the coupled response were obtained by Neely and Kim [19], assuming 1D fluid coupling. One criticism of the Neely and Kim model is that the active pressure has nothing to react off and is thus physically incomplete. The Neely and Kim model is a solid basis for the cochlear micromechanics and has been used and adapted by other researchers for the human cochlea, for example, by Elliott and Ku [22, 151]. The other important aspect in the cochlear micromechanics is structural longitudinal coupling in the BM and the TM. Mammano and Nobili [31] proposed a micromechanical model of the cochlea described by an integral equation. In the model, the shear motion between the TM and the RL was assumed as a function of the BM displacement and the way that the forces applied by OHCs amplify the BM motion is described as a cancellation of the fluid viscosity. They suggested that longitudinal elastic coupling due to the TM and RL is negligible as far as small BM amplitudes are involved. In a more recent paper [106], Meaud and Grosh found that viscoelastic longitudinal coupling in the BM and the TM is nonnegligible and the latter one plays a more important role in controlling the sharpness of the BM frequency response and the duration of the impulse response.

More detailed lumped-parameter micromechanical models have been proposed that have three degrees of freedom [32, 75] or even more. These clearly have even more parameters that have to be selected. Although it would be attractive to think that these parameters could be deduced from the dynamic behaviour of the physical OC, this is generally not possible. In practice the parameters are selected, as in the Neely and Kim model, to provide a response that appears reasonable, and a great deal of time can be taken up in such “tuning” of the many parameters. There are also so many parameters, that similar responses can be obtained with many different sets of parameters, and the selection problem is probably not unique. Also, the parameters selected to reproduce one aspect of cochlear behaviour; for example, spontaneous otoacoustic emissions [72] do not tend to work well in reproducing other aspects of behaviour, for example, distortion product otoacoustic emissions [128].

**Figure 24:**
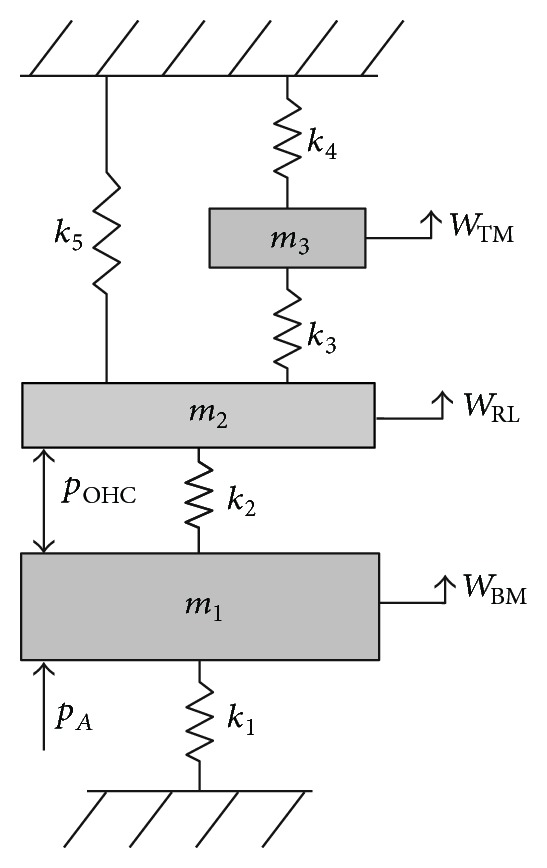
A lumped-parameter model of a cross-section of the physical arrangement of the cochlear partition. The transverse BM motion being driven both by the pressure difference in the fluid chambers and the pressure due to the OHC. The TM moves transversely and is radially driven by the OHC via the RL. The forces due to the radial motion can be resolved into equivalent transverse forces and the radial TM degree of freedom can be represented as an equivalent transverse degree of freedom.

Figure 24 shows an equivalent mechanical system of a cross-section of the cochlea, including the main components of the OC [21, 152, 153], as shown in Figure 1(b). In this model, the arch of Corti is assumed to pivot about its left bottom corner, which is attached to the BM and causes this to rotate as a more or less rigid body around this point [154]. Similarly, the reticular lamina is believed to rotate about the top vertex of the arch of Corti. The relative displacement of the OHC stereocilia is used in the model to recreate the force that simulates an active cochlea.

Three equations of motion can be used to describe the dynamic behaviour of the three-degree-of-freedom system, shown in Figure 24, as [21, 152, 153]

(51) PA−POHC  =m1w¨BM+k1wBM+k2(wBM−wRL),POHC=m2w¨RL+k2(wRL−wBM)+k3(wRL−wTM)+k5wRL,0=m3w¨TM−k3(wRL−wTM)+k4wTM,

where *m*_1_, *m*_2_, and *m*_3_ are the BM mass, the physical TM mass, and the transformed TM mass due to its shear motion, *k*_1_, *k*_2_, *k*_3_, and *k*_4_ are the BM stiffness, the OHC stiffness, the HB stiffness, and the shearing stiffness of the TM, respectively. *w*_BM_, *w*_TM_, and *w*_RL_ represent the displacement of the BM, the RL, and the equivalent shear motion of the TM. The damping can be incorporated in by defining a complex stiffness term.

**Figure 25:**
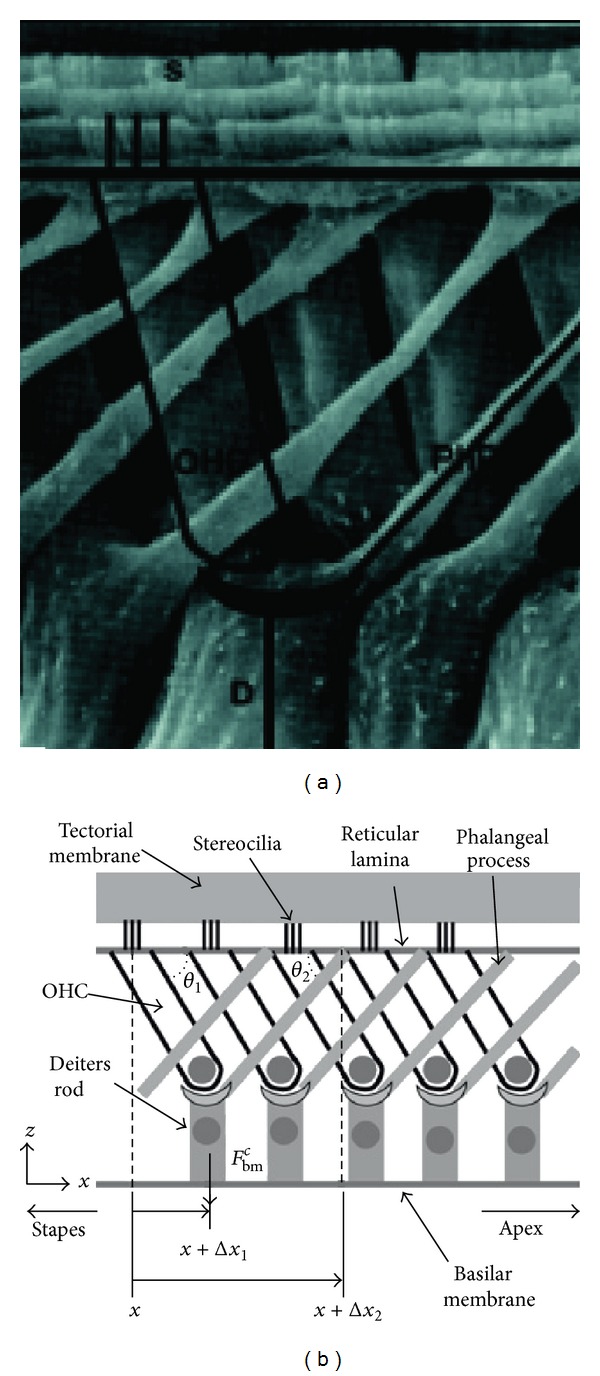
(a) Anatomical scan of a longitudinal view of the organ of Corti of a mole rat cochlea with a representative OHC, Deiters' rod (D), phalangeal process (PhP), and stereocilia bundle (S). (b) Schematic of the longitudinal view of the organ of Corti, showing the tilt of the OHCs based on (a) [160] (reprinted from Biophysical Journal, 100, Yoon et al., Feedforward and Feedbackward Amplification Model from Cochlear Cytoarchitecture: An Interspecies Comparison, 1–10, Copyright (2011), with permission from Elsevier).

Many researchers have sought to refine active cochlear models by including “feedforward” [32, 155] or “feedbackward” [139, 156] coupling between adjacent BM impedances. The term “feedforward” refers to the force, which is positive, provided by the OHCs being tilted apically toward the helicotrema and the term “feedbackward” refers to the force, which is negative, provided by the PhPs basally toward the stapes, as shown in Figure 25. In particular, the apical inclination of the OHCs has been shown to provide a spatial “feedforward” [110] effect that greatly enhances the wave amplitude near the characteristic place. This idea has been used in 1D, 2D [32, 157], and 3D [35, 158, 159] cochlear models. However, Yoon et al. [160] pointed out that the BM velocity simulation results from these models do not agree with those from* in vivo* experiments in phase lags. There is an excessive phase excursion and there is a shift in the best frequency between the passive and active models, which is about an octave in simulations but half octave in measurements.

#### 4.2.2. Three-Dimensional Models

Apart from those relatively simply formulated active cochlear models, many researchers have sought to refine model predictions by adding more degrees of freedom to the CP [20], expanding solutions into multiple spatial dimensions [86, 161]. The goal of these models is to include components of the cochlea to represent as realistically as possible the cochlea mechanics. Steele and Lim proposed a three-dimensional model of the guinea pig cochlea incorporating the viscous fluid effect, inner sulcus mechanics, feedforward, and also material variation along the cochlear length [158]. This model consists of two degrees of freedom, one for the flexing of the pectinate zone and one for the rocking of the arches. The phase-integral method, WKB, is used to calculate the solutions for the model and results show that a second travelling wave occurs due to the incorporation of the sulcus mechanics, though this has not been observed in auditory nerve responses. In order to simulate in the time domain to predict two-tone suppressions and harmonic distortion these nonlinear properties, Lim and Steele [35] proposed another three-dimensional model with the BM modelled as an orthotropic plate. Full three-dimensional models include varying details and geometrical complexities have also been built using the FE method by Kolston and Ashmore [86] and Böhnke and Arnold [85]. One of the attractive findings from the Kolston and Ashmore [86] model is that the TM and DC structural properties, especially the DC axial stiffness, play an important role in the OHC-BM feedback loop, which is due to the fact that the DC is the connection between the OHCs and the BM and the active force transmitted by the DC to the BM is determined by the axial stiffness. However, one limitation of their model is that the OHCs and DC are modelled as one-dimensional structures indicating they cannot offer impedance to radial fluid motion within the OC which is assumed to occur in the spaces between the OHCs in the real cochlea. Böhnke and Arnold [85] propose a finely detailed model to represent the OC, in which OHCs and DCs are modelled as 3D beams, to demonstrate the nonlinear mechanism in the cochlea. Although the model is only of 60 *μ*m length and cannot represent the whole cochlear structure, it still well presents the micromechanics of the OC and is practical for computing.

The high dimensional cochlear models with various details of the OC are ideally suited for studying the micromechanics of the cochlea. They have the common limitation that large numbers of degrees of freedom require massive computing time and a powerful computer. Solutions in the time domain are difficult because they require analysis at thousands of time steps.

## 5. Nonlinear Models

In a linear cochlear model, a sinusoidal pressure difference across the CP generates a sinusoidal BM velocity and so the relationship between them can be simply represented by an impedance function. In a nonlinear cochlear model, however, the waveform of BM velocity response to a sinusoid becomes distorted. Many efforts have been made to model the nonlinear behaviour of the cochlea, including the compressive growth at high sound levels, two-tone suppression, and DPOAEs [34, 36, 162–164]. The nonlinearity is typically obtained by defining nonlinear elements, such as nonlinear damping, nonlinear OHC force or pressure, and nonlinear geometry.

### 5.1. Nonlinear Functions

To model the nonlinear behaviour of the cochlea, we need to define nonlinear functions to describe the system. In general, the input, *u*, and output, *v*, of a system are related as

(52) $$\begin{array}{c} v=H\left(u\right). \end{array}$$

For a linear model, if *u* is multiplied by any constant factor, the output *v* will be scaled by exactly the same constant as

(53) $$\begin{array}{c} \beta v=H\left(\beta u\right). \end{array}$$

And, by superposition, for multiple inputs *u*_1_, *u*_2_,…, *u*_*n*_, the response to the sum of those inputs is equal to the sum of the responses to the individual inputs as

(54) $$\begin{array}{c} H\left(u_{1}+u_{2}+\cdots +u_{n}\right)=H\left(u_{1}\right)+H\left(u_{2}\right)+\cdots +H\left(u_{n}\right). \end{array}$$

A linear system cannot generate signal components that were not present in the stimulus spectrum, but any nonlinear system will produce harmonic distortion products in response to simple tonal stimuli, and more complex stimuli produce more complex distortion product spectra. To study cochlear nonlinear behaviour, a time domain analysis is generally necessary. To start the time domain analysis, all relevant system equations should be setup in the time domain using differentiation and integration wherever appropriate.

### 5.2. Nonlinear Damping

Generally, the relationship between the pressure difference, *p*, and BM acceleration, $\ddot{w}$, at different positions, *x*, along the cochlea can be expressed by the long-wave approximation as

(55) $$\begin{array}{c} \frac{\partial^{2}p\left(x\right)}{\partial x^{2}}=-\frac{2\rho }{h}\ddot{w}\left(x\right), \end{array}$$

where the BM dynamics are related to the pressure difference as

(56) $$\begin{array}{c} p\left(x\right)=m\left(x\right)\ddot{w}+r\left(x,\dot{w}\right)\dot{w}+s\left(x\right)w, \end{array}$$

in which mass is usually assumed to be constant.

Substituting (56) into (55) gives

(57) $$\begin{array}{c} {\left[r\left(x,\dot{w}\right)\dot{w}+m\left(x\right)\ddot{w}+s\left(x\right)w\right]}_{xx}=\frac{2\rho }{h}\ddot{w}\left(x\right), \end{array}$$

where [ ]_*xx*_ denotes double differential by *x*.

To solve this equation in the time domain, the first step is to transform it into two coupled ODEs and then the spatial differential equation can be transformed to a difference equation. The remaining ODEs in time can be treated separately. The second-order equations can also be rewritten as sets of first-order equations, which can be solved with the numerical methods such as the Runge-Kutta method [165].

Different researchers define different forms for the function *g* to represent the nonlinearity in their cochlear models. Kim et al. [166] studied the nonlinear behaviour of the cochlea using a 1D nonlinear model, which is represented by a nonlinear differential equation. The damping term in this differential equation is a function of velocity and “effective frequency” which depends on the input level. They assumed the characteristic frequency to be the limiting value of the effective frequency as the input amplitude approaches zero. Using this model they predicted the distortion products and other nonlinear phenomena in BM motion. The model behaves effectively linearly at low input levels and nonlinearly at high input levels. In a similar way, Hall [36] incorporated the nonlinearity in a transmission-line model of the cochlea by defining a nonlinear resistance and calculated the nonlinear BM response and distortion products in the spatial domain.

One famous model is called the Van der Pol oscillator. Basically, the oscillator is described by an ODE similar to one that describes a damped mass-spring system, but the damping term is an even nonlinear term, which has a negative value for small amplitudes, which would make the oscillator unstable. In his model, the damping term has a form of the quadratic (parabolic) function. Small amplitude negative damping specifies an oscillator that generates a limit cycle oscillation if undriven and uncoupled. The relative scaling of the active part is characterized by a parameter *ε*, which determines the major characteristics of the response. At high amplitudes, the damping is positive again and monotonically increasing (at least nondecreasing). For large amplitude, and also for large velocity *v*, the damping terms *r* is set to be proportional to *v* ^2^, which leads to a power of 1/3 dB/dB in the output-input level curve. It should be noted here that this symmetric nonlinearity can only generate odd-order harmonic and intermodulation distortion [167]. Following the van der Pol oscillator model, Duifhuis et al. [168] gave another form for the damping terms at position *x* as

(58) $$\begin{array}{c} r\left(x,v\right)=-r_{1}\left(x\right)+r_{2}\left(x\right)v^{2}, \end{array}$$

where *r*_1_ and *r*_2_ are related as the product *r*_2_/*r*_1_ *v* ^3^ is dimensionless.

Duifhuis also suggested that overlap of the responses is found in the Van der Pol oscillators in the frequency domain and also a decrease of selectivity. He proposed a modified Van der Pol oscillator model [167] with a general even-order nondecreasing damping term, which has a function that grew more slowly than quadratic and might even saturate as

(59) $$\begin{array}{c} r\left(x,v\right)=r_{0}\left(x\right)\left[\chi \frac{\sinh\left(\alpha v\right)}{\alpha v}-\frac{\gamma }{\cosh\left(\beta v\right)}\right], \end{array}$$

in which the hyperbolic sine function is approximately constant for small amplitudes and increases exponentially at large amplitudes.

**Figure 26:**
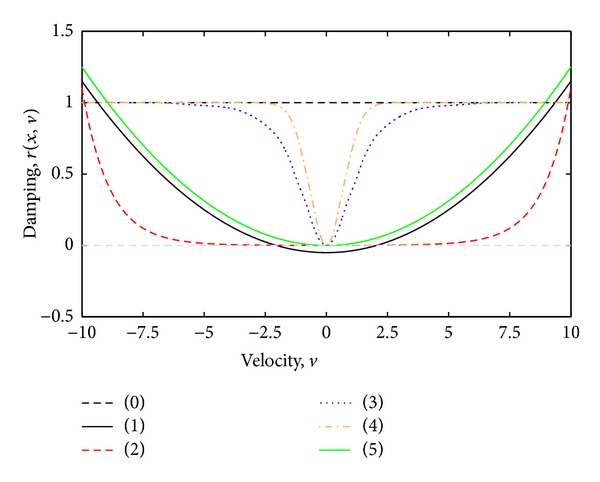
Scaled damping profiles as a function of velocity. (0) A linear profile is independent of velocity. (1) The modified VDP profile and the sinh⁡⁡(*x*)/(*x*) profile (2) continue to increase with increasing velocity. Profiles (3) and (4), (59), move from low values with low velocities to a saturation level with high velocities. The Hopf-profile (5) [209] can be considered as a version of the VDP profile, which is scaled so that its limit value for small deflections is exactly zero.

The nonlinear model proposed by Furst and Goldstein [38] was also very suited for time domain analysis. The *g*-function proposed by Furst and Goldstein contains a nonlinear damping profile, similar to the Van der Pol nonlinear profile, but lacking its characteristic near-zero negativity. An additional nonlinear correction is applied to the stiffness term as

(60) $$\begin{array}{c} g\left(x,t\right)=r\left(x,t\right)v\left(x,t\right)\left[1+av^{2}\left(x,t\right)\right]+\frac{Y\left(x,t\right)s\left(x,t\right)}{1+{\left|bv\left(x,t\right)\right|}^{\delta }}, \end{array}$$

where *a*, *b*, and *δ* are parameters. When proper middle ear coupling is incorporated into the model, it can be used to generate DPOAEs but cannot predict SOAEs or CEOAEs [169].  Figure 26 shows some examples of nonlinear damping profiles using different functions.

### 5.3. Nonlinear OHC Force and Geometrical Nonlinearity

Besides nonlinear damping in the single-degree-of-freedom model, researchers also developed various nonlinear force or pressure models to represent the effect of the OHCs motility in the more complicated micromechanical models in Section 4.2. Kanis and de Boer [33] developed a model to replicate the nonlinear behaviour of the cochlea. Their motivation was to provide insight into the mechanisms of cochlear nonlinearity with a simple model. The model is a long-wave model of the cochlea, 1D, containing a saturating pressure generator, located at the OHCs, which modifies the BM velocity via adding a nonlinear pressure to the pressure difference across the OC. To achieve that, they define the nonlinear pressure using the* hyperbolic tangent function* and solve the model with a “quasilinear” iterative method in frequency domain. Later [170], they compared the result with that from the time domain solution proposed by Diependaal et al. [39] and showed that the two methods match well for single-tone and two-tone stimulations.

Chadwick [34] treated the nonlinearity as a correction to a linear hydroelastic wave in his two-degree-of-freedom lumped-parameter model. The active force generated by the OHC was expressed as a function of TM-RL transverse displacement and the saturation of this force was represented by the hyperbolic tangent function. He used multiple scale approximation to express the equation of motion in a nonlinear homogeneous integrodifferential form and then related the active force function to the local wavenumber which is a function of the BM displacement. In this way, the nonlinearity in the model depends not only on the TM-RL displacement but also on the BM displacement. He also showed that the nonlinearity of the outer hair cell generates retrograde waves travelling backward toward the base.

Böhnke et al. [171] constructed a complex 3D FE human cochlear model including detailed OC structure to further analyse the active and nonlinear mechanics. In their model, the OC was modelled with finite length including 8 OHCs using the finite element method. Since only a section of the OC was considered so that the travelling wave was neglected in their model, the load due to surrounding lymphatic fluid was represented by a symmetrical loading on the OC. The nonlinearity was represented by defining a nonlinear OHC function, which expresses the OHC receptor potential as a function of the stereocilia displacement, using a* second-order Boltzmann function*. However, the effect of the nonlinearity was only roughly shown in terms of the distorted time signal.

Lim and Steele [35] developed a 3D uncoiled feedforward nonlinear cochlear model based on the WKB method. The model was built based on geometric and material properties from a chinchilla cochlea, in which the CP was modelled as an elastic orthotropic plate and viscosity of the cochlear fluid was also included. Fourier series expansions were used for an iterative procedure of solving the model. The model was used to predict BM response under different input levels, BM frequency response, BM velocity compression, distortion, and two-tone suppression. Comparison between numerical results and those from experiments shows a reasonable agreement. Following studies of OHCs mechanoelectrical transduction (MET), Liu and Neely [163] construct a nonlinear model to study DPOAEs. The receptor current that flows into an OHC is defined as a* nonlinear antisymmetric function* of the RL displacement and velocity.

Böhnke and Arnold [85] analysed the geometrical nonlinearity caused by the phalangeal processes using a 3D FE human cochlear model including detailed elements within the OC, such as OHCs, RL, and Deiters' cells. They considered large deflections, large rotations but small strains, and also the stiffening effect on the structure due to its state of stress. These two types of geometrical nonlinearities in the Deiters' cells were modelled using a thin elastic beam with low modulus of elasticity. In their model, an external force due to two-tone stimulation is applied on top of the RL. This leads to a displacement of the OHC between the RL and the DC. When the model is linear, the amplitude spectrum of output displacement clearly shows two peaks corresponding to the two driving frequencies. When the geometrical nonlinearity is considered, however, distortion components appear in the spectrum.

Comparing 2D and 3D nonlinear cochlear models, Diependaal concludes [172] that the 2D response is close to the 3D solution but that the 1D solution deviates considerably from the multidimensional solutions. However, he points out that response from the 2D passive model with realistic values of the parameters may deviate significantly from the 3D response. So the dimensionality of a cochlear model depends on how much output one wants to obtain from the simulation. If one is only interested in cochlear macromechanics, a 1D approach is often satisfactory. However, if one wants information about interaction within the OC, cochlear micromechanics, a more detailed model, a 2D, sometimes a 3D, is required.

### 5.4. Solving Nonlinear Equations

In practice, it is usually impossible to analyse the nonlinear equations analytically, which requires numerical methods of time domain analysis or of linear approximation and perturbation techniques [39, 40, 166, 173–176]. In some cases, the perturbation approach is an alternative to the numerical analysis in the time domain. As long as the nonlinear effects are relatively small in the model, the behaviour can be approximated by a set of linear equations, and then a number of iteration steps adjust for the relatively small nonlinear deviations. Taylor expansion and Fourier series have been used to solve the nonlinear models in the time domain. It may require many steps before a proper stable solution is approached, and limitation to a few steps can lead to disastrous errors. This type of approach starts with a linear approximation of the solution and then tries to improve the solution by using iteration steps. At each step, the number of terms in the expansion is increased, up to the point where the remaining error is deemed acceptable.

The state space approach is inherently set in the time-domain and can express the dynamics of a system as a set of coupled first-order differential equations and arranged in vector matrix form [22] as

(61) $$\begin{array}{c} \dot{x}\left(t\right)=Ax\left(t\right)+Bu\left(t\right),\\ y\left(t\right)=Cx\left(t\right)+Du\left(t\right), \end{array}$$

where **x**(*t*) is the *m* × 1 vector of the states of a *m*th order cochlear model, **A** is the *m* × *m* system matrix that contains the mechanics of the cochlear model, **B** is the *m* × *r* input matrix that scales the *r* inputs to the model, **u**(*t*) is the *r* × 1 vector composed of the model input functions, **y**(*t*) is the *p* × 1 vector composed of the defined *p* outputs, **C** is the *p* × *m* output matrix that selects the output states of the model, and **D** is the *p* × *r* feed-through matrix that transmit the input directly to the output, where *m*, *r*, and *p* are integer values defining the dimensions of the vectors and matrices. The stability of the state space system can be determined by calculating the eigenvalues of the system matrix, **A** ([22, 72, 151]).

Although there are kinds of active nonlinear cochlear models built by different numerical approaches, most of them can be implemented in the state space formalism [177]. For the cochlear models [39, 40, 155, 170, 178, 179] which have one-degree-of-freedom, there are two states, namely, BM displacement and velocity. For the cochlear models with two-degree-of-freedom [19, 22, 32, 77, 80, 180], there are four state variables associated with the displacement and velocity of the BM and TM. In this way, the coupled cochlear model can be solved in the time domain using a Runge-Kutta algorithm with variable internal step size [39, 165, 176]. Epp et al. [165] adopted a fourth-order Runge-Kutta method with a computational frequency of 400 kHz to solve their nonlinear active cochlear model, in which the nonlinearity was described by a combination of velocity dependent damping and feedback stiffness defined by a double sigmoidal function.

## 6. Electrical Coupling

Mechanical vibrations in the cochlea are generated by a pressure input stimulus having a large dynamic range. These vibrations activate the hair cells (sensory organs) in the cochlea. Hair cells detect these vibrations, reduce their dynamic range, and encode them to a form that the nervous system can interpret. These processes are only possible because of electrical activities inside the cochlea. The following is a brief overview of these electrical activities, the electrical properties of the cochlea, and the mutual interaction between electrical and mechanical parts in the cochlea, as well as models of these phenomena.

### 6.1. Electrical Properties of the Cochlea

Recall from Section 1.2 (Anatomy of the Cochlea) that the cochlea is made up of three compartments which are filled with two fluids called endolymph and perilymph. The endolymph, which fills the scala media, has a unique ion content which makes it more electrically positive than both the other fluid (perilymph) and intracellular potentials. These differences in potential levels produce standing flows of ions through various structures of the cochlea which maintain steady state potentials and currents in the cochlea. It is noteworthy to mention that the unique ion content of the endolymph is maintained by the electrogenic pumping of potassium by stria vascularis [181]. Note that even though purely mechanical models of the cochlea as explained so far can provide much information about the cochlea function, incorporating a detailed electrical model of the cochlea can lead to better understanding of the functions of hair cells and consequently the cochlear functions.

Vibrations of the basilar membrane deflect the stereocilia and modulate these flows of ions. Deflection of the stereocilia opens and closes pores known as MET channels, and due to the potential difference between the perilymph, endolymph, and the intracellular potential, the opening and closing of the MET channels changes the inflow of ions which results in activation of the hair cells. For investigating the properties and effects of these standing and alternating flows of ions, the cochlea can be modelled as a network of biological resistances, capacitances, and voltage and current sources, that is, an electrical model.

#### 6.1.1. Inner and Outer Hair Cells

As previously mentioned, the organ of Corti consists of sensory receptors called the IHC and OHC. The IHC transduces mechanical vibration into neural stimulation which is sent to the brain for interpretation. The OHC nonlinearly amplifies the small basilar membrane motions, and this action consequently enhances the sensitivity of IHC to weak stimuli and compresses high-level stimuli. Thus the operation of the OHCs enormously increases the dynamic range of hearing [182–184].

At least the longest stereocilia of the OHCs are imbedded in the tectorial membrane, and hence the MET channels of the OHCs appear to be sensitive to the relative displacement of the basilar membrane and tectorial membrane [185]. This observation is actually at variance with some observed behaviors of the cochlea, and several explanations have been proposed for the discrepancy [186]. The stereocilia of IHCs are sensitive to the velocity of the radial flow of endolymph.

The hair cells connect to the nervous system via the eighth cranial nerve [187]. The interactions between hair cells and the nervous system in response to sound stimulus produce the whole-nerve or compound action potential of the auditory nerve which is recordable from round window electrodes [184] or at their generation sites. The innervation of the organ of Corti indicates that the IHCs transmit electrical signals to afferent fibers, and thus IHCs seem to be purely sensory while the purpose of afferent connections of the OHCs still remains unclear [184].

The IHCs and OHCs are the primary elements of the electrical lumped model and can be individually divided into apical and basolateral parts. Each part can be modelled by membrane capacitances, variable resistances, and voltage sources.

#### 6.1.2. Mechanical Effects of the OHC

The OHC in the mammalian cochlea is thought to use both somatic electromotility and hair bundle electromotility to provide mechanical active amplification [183].

*Hair Bundle Motility.* The mechanosensory organelles which protrude from the apical surface of the hair cells comprise the hair bundle [188]. Hair bundle motility is considered by some researchers to have an effective amplification property in mammalian hair cells [189]. Deflection of the hair bundle changes the stereociliary calcium ion concentration and causes the hair bundle (using the myosin motor protein) to spring back, opposing the stimulus [182]. The hair bundle force is linked to the displacement of the hair bundle and the probability of the opening of the MET channels [190]. The hair bundle electromotility mechanism was put forward to explain amplification at high frequencies for which the membrane time constant was thought to restrict OHC amplification [191].

More recent measurements have shown that the membrane time constant does not limit OHC amplification. The membrane time constant is approximately one order of magnitude smaller than what was previously reported [192, 193]. Experimental data indicates that the energy contribution of somatic electromotility of the OHC is much larger than that of hair bundle motility and is therefore the primary amplification mechanism of mammalian OHC [194–197], and this conclusion has been supported by comprehensive cochlear models [198]. Hair bundle motility may be more significant in the cochlear apex than near the base [198]. The force intensity and purpose of the hair bundle electromotility mechanism in the mammalian cochlea are disputed and still require more investigation [183, 189].

*Somatic Motility.* Change to the length of the OHC is an active mechanism which makes the mammalian cochlea remarkably sensitive and precisely frequency sensitive. The OHC length depends on the hair cell membrane potential which in turn depends on the current flowing through the MET channel. Both of these effects are nonlinear. The MET channel current can be described as a Boltzmann function of hair bundle displacement [199] and this is the primary source of nonlinearity.

By using some simplifications, it can be shown that the ratio of OHC length change to the membrane charge, *Q*, movement is approximately constant and the OHC can be modelled as a piezoelectric material [200]. A capacitance can be calculated as the first derivative of *Q* with respect to cell membrane voltage *V* [186]. Although this effect is also nonlinear, the relationship is usually considered to be approximately linear. These relations couple electrical property of the OHC soma with the OHC mechanics and represent the OHC somatic motility [193].

#### 6.1.3. Organ of Corti

The electrical properties of the organ of Corti without hair cells are similar to other biological tissues of the human body and can be modelled as a passive electrical network. However, the existence of the hair cells gives special electrical properties to this sensory epithelium and affects its mechanical behaviours. The mutual interactions between mechanical and electrical parts of the organ of Corti have significant effects on the cochlear function and must be incorporated in a realistic cochlear model.

**Figure 27:**
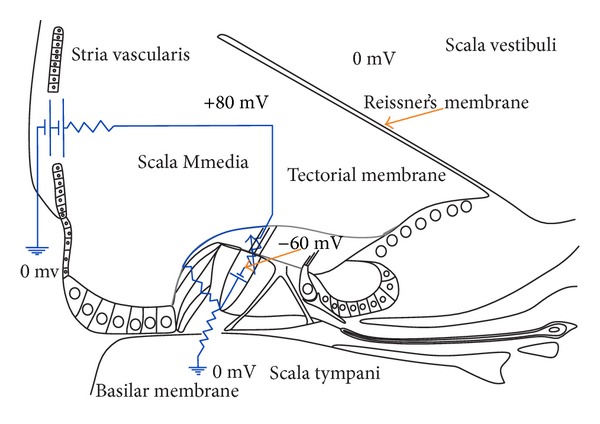
Battery or variable resistance model; +80 mv and −60 mV are the endolymph and intercellular potential, respectively. From [201] with permission.

**Figure 28:**
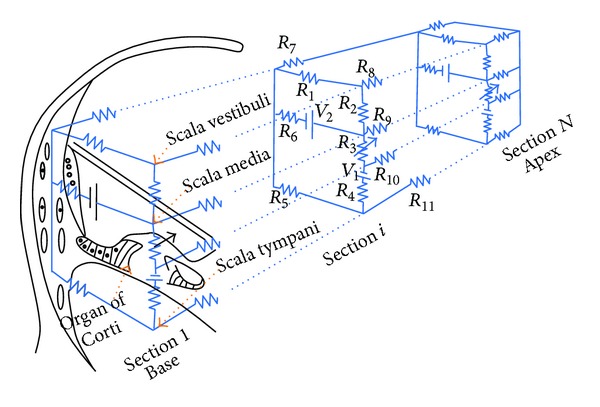
Strelioff's network model [202]. In this model, the cochlea is considered in cross-section slices. Each slice has six transverse resistors (*R*_1_ to *R*_6_) and five longitudinal resistors (*R*_7_ to *R*_11_). The parameter values of this model have been widely used by other investigators in this area (reprinted with permission from Journal of the Acoustical Society of America, 54, D. Strelioff, A Computer Simulation of the Generation and Distribution of Cochlear Potentials, 620–629, Copyright (1973), Acoustic Society of America).

The battery or variable resistance model by Davis [201] is an initial attempt to model the electrical network properties and the distribution of potentials in the cochlea. In this model, the resting potentials of the cochlea have been modelled by two batteries: a primary battery in the hair cells and an accessory battery in the stria vascularis. The MET channels are modelled by variable electrical resistors. Accordingly, the current through the hair cells is modulated by changing electrical resistances resulting from cilia deflection, as shown in Figure 27. Strelioff [202] suggested a network model of the resistors and batteries to simulate the generation and distribution of the cochlear potentials, as shown in Figure 28. The results of this model were in agreement with previous physiological findings.

In [203, 204] electrical properties of the organ of Corti have been investigated. The electrical configuration component values of the resulting detailed model for a radial section of the organ of Corti have been determined heuristically based on actual measurements of potentials inside the cochlea. Some of these parameter values have been revised later by other researchers [192].

#### 6.1.4. Mechanical-Electrical Models

Even though electrical coupling has been rarely amalgamated into the cochlear models, some works can be seen in the literature.

In Ramamoorthy et al. [21], a model has been proposed which integrates the electrical, mechanical, and acoustical elements of the cochlea. This model provides a framework to successfully predict and reproduce the response of the cochlea to acoustical stimulus comparable to experimental data. Nonlinear characteristic of the MET channel and hair bundle motility have not been considered in this model. A notable observation from this model is that longitudinal electrical coupling actually sharpens the mechanical response. This effect is also reported in Meaud and Grosh [198] and is in part the motivation for the models of Iwasa and Sul [205] and Dimitriadis and Chadwick [206]. Whether this effect is significant in a nonlinear cochlea is not known.

Nonlinear saturation behaviour of the MET channel has been incorporated in Liu and Neely [163] to explore distortion product otoacoustic emission. In this model the longitudinal electrical connection in the organ of Corti and the hair bundle motility have been neglected. The model of Nam and Fettiplace [183, 193] has used a mechanical model along with electrical coupling to investigate the effects of the hair bundle motility and the cochlear amplifier in high and low auditory frequencies.

**Figure 29:**
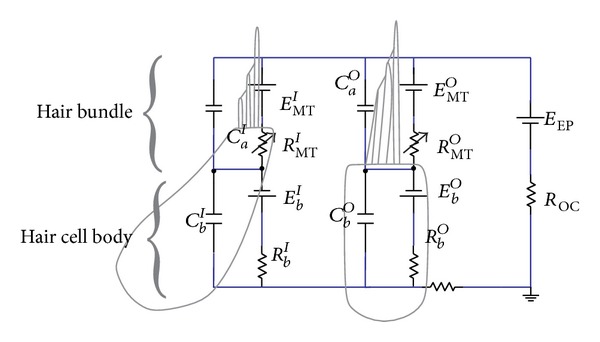
A circuit diagram for the inner and outer hair cells. Superscripts *I* and *O* denote inner and outer hair cells. Subscripts MT and *a* and *b* indicate the MET channels and apical and basolateral parts of the hair cells, respectively. This model configuration or similar versions of it can be seen in Dimitridias and Chadwick [206]; Iwasa and Sul [205]; Dallos [203, 204]; Mistrík et al. [210]; Johnson et al. [192]; Cheatham et al. [211] just to cite a few. Mistrík et al. [210] has used this electrical model with longitudinal resistances to model the current flow in a model of the cochlea, as shown in Figure 30.

**Figure 30:**
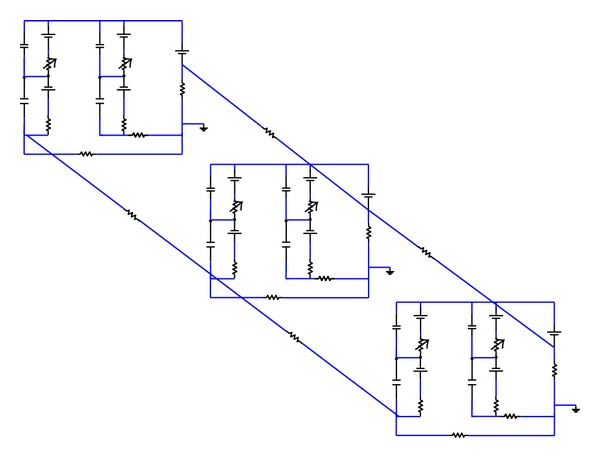
The electrical network model of the organ of Corti [210]. This model is made up of the circuit of together with longitudinal electrical coupling.

**Figure 31:**
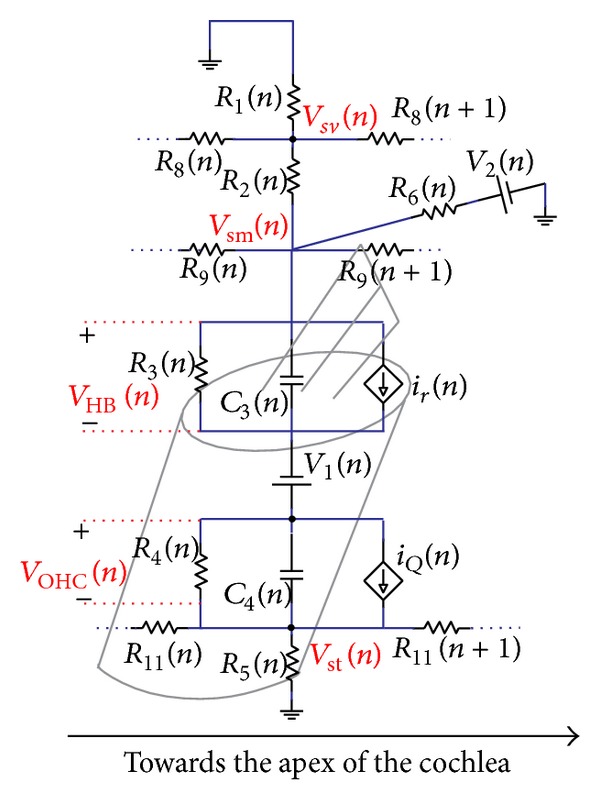
The electrical network model of the organ of Corti with dependent current sources instead of variable resistors [21, 212]. *i*_*r*_ and *i*_*Q*_ are the dependent current source for apical and basal surfaces, respectively. *R*_3_ and *C*_3_ model the apical resistance and capacitance. *R*_4_ and *C*_4_ model basolateral resistance and capacitance. *V*_HB_ and *V*_OHC_ are the potentials of apical and basal surface of the outer hair cell, respectively. The longitudinal electrical coupling is similar to Strelioff [202], as shown in Figure 28.

**Figure 32:**
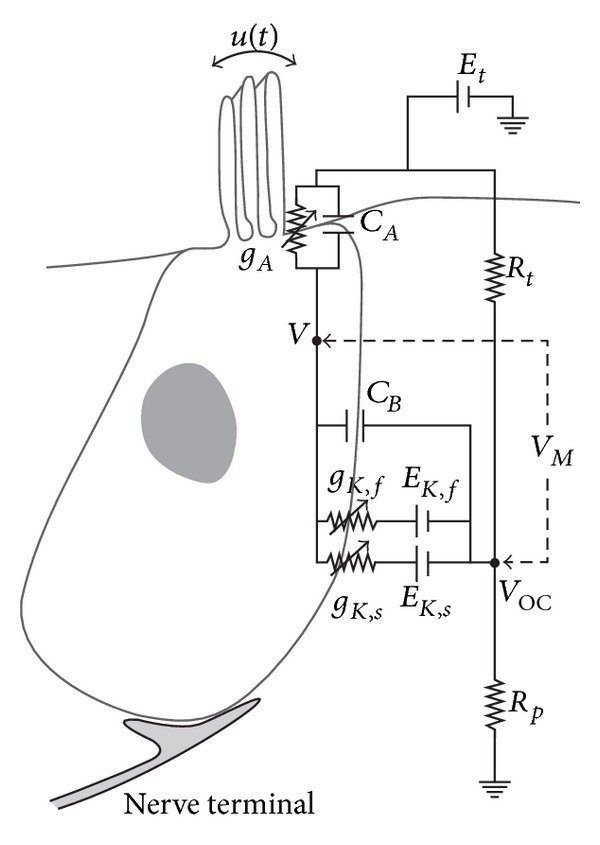
An electrical model of the inner hair cell [208]. In this model, the apical conductance *g*_*A*_ is a function of the IHC stereocilia displacement, *u*(*t*). *C*_*A*_ and *C*_*B*_ model the apical and basilar membrane of the IHC. *R*_*t*_ and *R*_*p*_ are epithelium resistances. *V*_M_ is membrane potential. *g*_*K*,*f*_ and *g*_*K*,*s*_ are fast and slow basolateral resistances. *E*_*K*,*f*_ and *E*_*K*,*s*_ represent potentiator associated with fast and slow resistances. From [208] with permission of Journal of the Association for Research in Otolaryngology.

Electrical properties of hair cells* in vitro* and* in vivo* have been thoroughly examined, and very sophisticated models with detailed ion channels can be seen in the literature [207]. A simple model of the IHC and OHC above is presented in Figure 29. This model or slightly different versions of it have been widely used in the area of modelling the distribution of the cochlear biopotentials and their effects.  Figure 30 illustrates this model with longitudinal coupling. Longitudinal coupling was proposed by Dimitridias and Chadwick [206] as a mechanism for nonlocal sensing in the operation of the CA.  Figure 31 shows an equivalent configuration for the imbedded OHCs with dependent current sources instead of variable resistors. A detailed circuit model of the IHC is presented in Figure 32. This model is used to investigate contribution of the IHCs to auditory compression [208].

### 6.2. Responses to Stimulus

**Figure 33:**
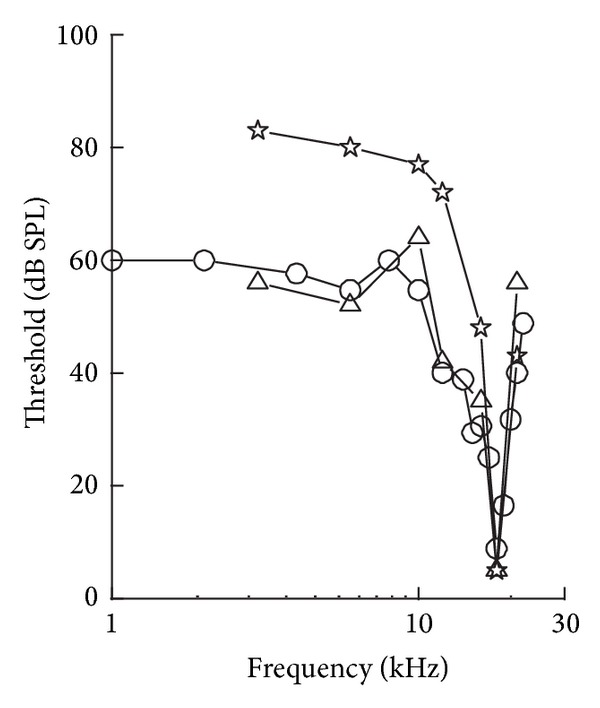
A comparison between the hair cells and basilar membrane tuning curves. The tuning curves are for IHC (○), OHC (△), and basilar membrane displacement (*☆*). From [184] with the permission of Elsevier.

**Figure 34:**
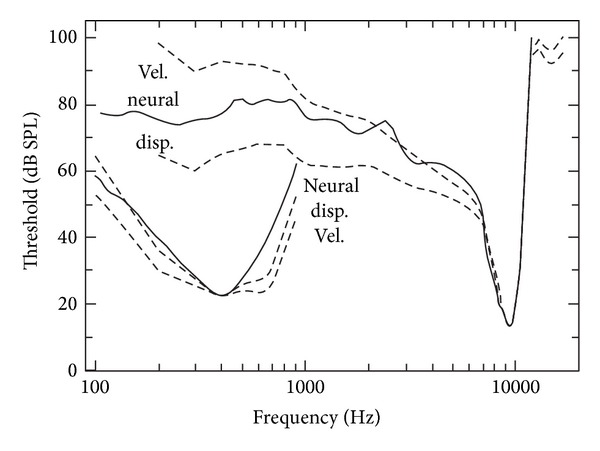
A comparison between the neural and basilar membrane displacement/velocity tuning curves. From [184] with the permission of Elsevier.

Experimental recordings from the cochlea show that the cochlear amplifier results in sharply tuned cochlear mechanical responses. These tuning curves indicate that auditory nerve excitation fully matches mechanical tuning [184, 213]. The tuning curves of the IHC and OHC potentials and the basilar membrane are compared in Figure 33. The basilar membrane velocity and displacement are reported in Figure 34. There is a tuning discrepancy between the aforementioned tuning curves and that of potentials recorded from the cochlea (the cochlea microphonic) which is discussed in Section 6.3.2.

### 6.3. Application

Using realistic electrical coupling in cochlear models will facilitate opportunities to gain further information about mechanical and electrical interactions.

#### 6.3.1. Neural Stimulation

Modelling the voltage distribution in the cochlea not only helps to understand cochlear function but also may be used to design new strategies for delivering acoustical signal information to auditory nerves which can be used in cochlear implants [214].

In cochlear implants the number of physical electrodes is limited to 12–22 in contemporary devices. This limited number of electrodes can only stimulate a small number of fairly broad fixed regions along the cochlea. Frequency resolution can be improved by using virtual channel techniques in which additional places can be stimulated by the available electrodes [215].

Two techniques have been developed to create a virtual channel. The current steering technique uses superposition of the electrical fields of two simultaneously activated electrodes to trigger the intermediate auditory neurons. In this approach, independent current sources are required. Another technique uses fast consecutive pulses to activate two electrodes. The impedance between these two electrodes completes the circuit and results in the stimulation of neurons between the electrodes [215].

#### 6.3.2. Cochlear Microphonic

Deformations of the BM cause deflection of the stereocilia which open and close the MET channels. This alternation causes a varying electrical current through the biological resistances and capacitances in the organ of Corti and generates the potentials both inside and outside of the hair cells. This potential outside of the hair cells is referred to as the cochlear microphonic (CM). The CM was first observed by Wever and Bray in cats [216, 217]. Adrian used the term cochlear microphonic to describe this signal [218, 219].

The cochlear microphonic is a by-product of the cochlear activities in response to a sound stimulus. The CM can be observed by placing an electrode on the surface of the cochlea [194] or with glass micropipette electrodes inserted in the scala media [220]. The CM can also be obtained as a far field signal which can be recorded noninvasively by placing electrodes inside the ear canal or invasively by placing electrodes on or close to the round window membrane [184, 221]. This potential can be used to assess the MET current of outer hair cells ([222–224]) checking the biological effects of infrasound on the human auditory system [225, 226], diagnosing Ménière's disease [227], and diagnosis of auditory neuropathy spectrum disorder [228].

The CM is mainly generated by the OHC [229] and electrical coupling models can reveal much information about the CM and its longitudinal distribution. The effect of the CM on nearby OHCs has been considered to be significant in the operation of the CA [205, 206]. As mentioned earlier, the cochlear amplifier causes the BM tuning curves to be sharply tuned at the characteristic frequency. However, the CM, which results from the BM vibration, has broad tuning curves [220, 230]. Furthermore, in some recordings and models of the CM, some notches can be observed in tuning curves of the CM [212, 230]. It is likely that the CM generated at the best place is reduced by interference with that generated at other locations ([230, 231]) and the observable notches may result from this cancellation. The phase difference between hair bundle and the OHC soma has also been suggested as another contributing factor to the broadness of CM tuning curves [232].

Integrated mechanical and electrical models have the potential to shed light on the source of these discrepancies and notches.

## 7. Conclusions

### 7.1. Highlights of Cochlear Modelling

#### 7.1.1. Data Requirement

Modelling work largely depends on available data from experimental measurements, but material properties of some components in the cochlea are difficult or impossible to obtain, especially for humans. Assumptions and data fitting are always used in modelling the cochlea, especially for the models of cochlear micromechanics. Great effort has been put into finding reasonable values for some modelling parameter, but unfortunately, many others are still largely empirical, making some models difficult to validate.

#### 7.1.2. Nonlinearity

The sources of the nonlinearities in the cochlear amplifier are still not well identified. Possible sources include material, geometrical (the dependence of the stiffness on the displacement), and state-dependent nonlinearities of mechanical structures; the nonlinear mechanoelectrical transduction process of auditory hair cells; and nonlinear neural coding of information, for example, rate intensity functions. A mixture of all these nonlinear factors is effective* in vivo* and it is difficult to distinguish the separate contributions [171].

The* Hyperbolic tangent function* was used to saturate the feedback force in early nonlinear cochlear models [22, 33, 34]. The disadvantage of the hyperbolic tangent function is the incapability of generating the even-order harmonics observed in experiments [233]. Later, the* Boltzmann function* was used in nonlinear cochlear models by many researchers [72, 171], since it is similar to measured input-output characteristics of OHCs in isolation [234, 235].

Another important area related to nonlinearity is the method of solving the models in the time domain. Ku [72] found that the simulation time of a nonlinear state space cochlear model can be reduced by fourfold without decreasing the accuracy by fine-tuning the error tolerances for each individual state. Bertaccini and Sisto [236] suggested that a hybrid direct-iterative solver is faster than standard sparse direct solvers for models in which the system matrix is data dependent.

An alternative approach for implementing a model in the time domain is to use a circuit analogy of the model, and a powerful nonlinear circuit simulator such as SPICE (simulation program with integrated circuit emphasis) to solve it [237].

#### 7.1.3. Cochlear Micromechanics

The discussion of multimode motion in the OC has been raised by some researchers [57, 102, 143]. This requires a complex cochlear model with detailed OC to investigate different coupling mechanisms, such as phalangeal processes (PhPs) between OHCs. Material properties, endolymph viscosity, and boundary conditions also need to be carefully considered in order to replicate experimental measurements.

#### 7.1.4. Modelling Damaged Cochlea

Besides replicating experimental finding in laboratory animals, the other important goal of modelling the mammalian (human) cochlea is to predict hearing defects [238, 239] or the effects of cochlear implants. Although there is still a great deal of basic research to be done on the hearing system, it is important for cochlear modellers to consider potential application of the models in the clinical area. Some preliminary work has been done to predict the effects of a cochlear implant on the BM response [240] and fluid coupling [70]. It is of great importance to predict the insertion position of the CI and interaction between the CI and the BM using relatively simple models to provide information for clinicians.

### 7.2. Open Issues and Debates

#### 7.2.1. Match and Prediction

One goal of modelling cochlear mechanics is to replicate results observed in experiments. A complete quantitative cochlear model is currently not feasible, since it requires extensive geometrical and material properties, which are difficult to measure. In most cochlear models, parameters have to be tuned to match observations from experiments, which is time consuming. It is quite often that good agreement only occurs for a specific experimental observation but cannot replicate all cochlear behaviours.

Another objective of modelling the cochlea is to predict phenomena yet to be observed. If the model works well on replicating certain behaviour of the cochlea, it is important to test predictions from the model, which have not been observed in practice, in new experiments.

#### 7.2.2. Fast versus Slow Waves

Many authors [61, 241] decompose the intracochlear pressure into two modes, the fast-mode, due to the mean pressure in the fluid chambers, and the slow-mode, due to the pressure difference. In this hypothesis, the fast-mode is believed to play a dominant role for frequencies higher than the best frequency and can cause an in-phase motion of the cochlear partition (much smaller than that caused by the slow-mode), which will not excite the cochlear amplification process [241]. However, the slow-mode will cause an antiphase motion in the cochlear partition, which excites the cochlear amplification process. The notch in measured intracochlear pressure found in some experiments is believed to be due to cancellation between the fast and slow modes. A notch is also obtained, however, in 3D cochlear models, which is believed to be the consequence of transition between the travelling wave mode and a higher-order mode [56, 101]. Elliott et al. [57] use a wave approach to decompose the results from the full FE analysis to show the contribution of each wave which suggests that a higher-order fluid wave (local evanescent wave) starts to dominant the overall response after the characteristic place.

#### 7.2.3. Scale of Modelling

A multiscale model of the cochlea is always desirable, since it can incorporate detailed components in the cochlear partition. The question is how deep a model needs to be. A 2-DOF model can reasonably well represent the passive, active, and nonlinear behaviours of the cochlea. Detailed micromechanics, however, are ignored. To understand functions of each element, such as the RL, OHCs, IHCs, BM, and TM, in the OC, a detailed model including these elements is needed. Moreover, to explore the mechanism of mechanoelectrical transduction, which is believed to be the reason for cochlear active process, modelling on a scale down to nanometre may be required to show the molecular details of the myosin motors that maintain the tension in the tip links that connect the individual stereocilia within the hair bundle.

The argument is whether a model should cover every scale of the cochlea, namely, from millimetre for the SV and ST down to nanometre for tip-links, or, different scales could be considered separately and incorporate each individual response together to give an overall response. The former would require an extremely fine mesh for those tiny components, which leads to enormous number of elements that may cause convergence and computation difficulties.

Nonlinearity is an elaborate feature in the cochlea and could be modelled by different mechanisms, such as nonlinear damping, nonlinear OHC force, or nonlinear geometry. Different nonlinear mechanisms need different scales of modelling and it is difficult to say which scale is appropriate in any given situation.

#### 7.2.4. Somatic versus Bundle Motility for OHCs

In the mammalian cochlea, the amplification is a nonlinear active process providing extraordinary sensitivity and selectivity along with a large dynamic range and sharp frequency tuning. Although it is generally agreed that the amplification results are from active force generated by hair cells, there is a debate about the cellular processes behind the nonlinear amplification. One suggestion is that the outer hair cells electromotility underlies the cochlear amplification and another is that it is due to the active hair bundle motility.

The OHC electromotility is supported by a somatic motor in the OHCs body, which has the ability to elongate or contract axially, due to changes in membrane potential, and could provide positive feedback to reduce viscous damping and provide active amplification. Santos-Sacchi et al. [196] found fully reversible processes between alterations in OHCs electromechanical activity and cochlear amplification and modulating chloride activity* in vitro* and* in vivo*, which proved that OHCs motility is crucial for cochlear amplification. However, it has been also shown in frog and turtle that spontaneous movements of hair bundles endow them with a nonlinear response with increased sensitivity that could be the basis of amplification [191, 242, 243]. Sul and Iwasa [197] used a theoretical hair bundle model to study the effectiveness of hair bundle motility in the cochlear amplification, in which they assumed that hair bundle energy is sufficient to counteract viscous drag in the subtectorial space.

A finite element model of the organ of Corti, in which the fluid loading and fluid longitudinal coupling were excluded, was used by Nam and Fettiplace [183] to analyse the two mechanisms at both basal and apical end of the cochlea and found that they could induce comparable BM motion but differ in the polarity of their feedback on hair bundle position. Maoiléidigh and Jülicher [244] proposed a cross-sectional model of the cochlear partition, in which the TM, HB, RL, and BM were assumed to be rigid beam and suggested that the properties of the cochlear amplifier could be a result of the combination of both hair bundle motility and electromotility in an integrated system that couples these processes through the geometric arrangement of hair cells embedded in the cochlear partition. Following these researches, Meaud and Grosh [198] constructed a global mechanical-electrical-acoustical model of the guinea pig cochlea and found that the active HB motility alone is not sufficient to provide energy for high frequency amplification, but the somatic motility can overcome the basolateral membrane resistor-capacitor and provide sufficient mechanical energy for amplification in the basal regions. As suggested by Santos-Sacchi et al. [196], the OHC lateral membrane mechanical activity may occur for the mammal to supplement an exist amplification system as an extra boost.

#### 7.2.5. Difference between Mechanisms in Base and Apex

It is still a particular challenge to model the mechanism of the cochlea in the apex, since only a few experimental measurements are available from* in vivo* cochleae at this position. Generally, the active amplification near the cochlear apex behaves differently from that observed near the cochlear base.

Measurements show that there is a strong compressive nonlinearity in the cochlear basal region and that hair bundle and BM displacements are both amplified. However, the amplification in the cochlear apical region is somewhat unidirectional and only the hair bundle displacement is amplified [245]. Another difference between the apex and the base is the direction of the BM displacement and velocity when the IHC has a maximum excitation. At the apex, the maximum IHC excitation is found when the BM is between maximum displacement and velocity toward scala vestibuli. However, at the base, the excitation is with velocity toward scala tympani. Steel and Puria [246] pointed out that the phase of IHC excitation, related to the tension in the tip link of the tallest stereocilia of the IHC, can “have any phase,” particularly depending on the elastic properties of the overlying TM.

In conclusion, then, cochlear models are still limited by both a lack of detail in the models and experimental data on material properties and* in vivo* response for validation. Although significant advances have been made on both fronts in the last few decades, our understanding is far from complete.

#### 7.2.6. Backward BM Travelling versus Compression Wave Theory of OAEs

OAEs were initially observed by Kemp more than three decades ago [7]. Ever since then the reverse propagation mechanism of OAEs has been intensively studied and is still a subject of debate [247, 248]. Two opposing hypotheses have been put forward to explain backward propagation. According to the slow travelling wave hypothesis, the vibration backpropagates as a travelling wave using the BM as a medium [248–251], while in the other one, OAE exits the cochlea by a fast compression wave in the cochlear fluid [247, 252–257]. A wide variety of experiments and models have been devised for demonstrating the validity of both hypotheses and it has been shown that the original results of He et al. [258] can be reproduced in models without fast waves [259, 260]. However, in spite of all these attempts, the exact mechanism of reverse propagation still remains unclear.

#### 7.2.7. The Source of the SOAEs

SOAEs are one of the major classes of OAEs which can be detected in the ear canal without any acoustic stimulus. Existence of these emissions is an explicit manifestation of the active mechanism in the cochlea [261, 262]. However, there remains some discussion about the source of SOAEs. There are two different theories that have been established to explore the origin of SOAE. The global standing-wave model proposes that SOAEs are produced by coherent reflections between an impedance mismatch at the middle ear and perturbations in the mechanics of the cochlea. The SOAE amplitude is actively maintained and stabilized by the cochlear amplifier [263]. This mechanism appears to be the dominant one in mammals. The second model, known as the local oscillator model, suggests that the active elements inside the cochlea independently cause local oscillation [264–266] which may explain similar effects in other animals.

#### 7.2.8. Nonclassical Models and Longitudinal Coupling

In a “*classical*” model of the cochlea, the dynamics of the cochlear partition are described by a* local* parameter [267], whereas “*nonclassical*” models introduce some form of longitudinal coupling. Naidu and Mountain studied the longitudinal coupling in the basilar membrane in the excised gerbil cochlea and suggested that the cells of the organ of Corti increase the overall coupling exhibited by the BM and the longitudinal coupling should not be neglected in the region of characteristic place [268]. Jaffer et al. [269] detailed a one-dimensional model of the cochlea, in which longitudinal elasticity element was used to represent the aggregate mechanical effect of the longitudinally connective tissues in the organ of Corti, and showed that longitudinal elastic dynamics is weak but not negligible and exhibits a cubic nonlinearity. Also other types of longitudinal coupling exist in the cochlea including fluid coupling through sulcus and organ tunnel, tectorial membrane elasticity [107, 270] longitudinal electrical coupling between the hair cells [20] and the feedforward action of the outer hair cells [12, 110].

Ghaffari et al. [109, 270] showed ability of the TM to support traveling wave which is similar to that of the BM near the best place and suggested that the cochlear micromechanics should be treated as a global process involving significant longitudinal distances. Following this, the longitudinal coupling due to the TM started to be included in cochlear models [106, 183]. Meaud and Grosh [106] pointed out that TM longitudinal coupling has a more significant effect than BM longitudinal coupling and allowed higher stable gains for the cochlear amplifier.

Apart from the 1D fluid coupling accounted for in most models, there are many other forms fluid and mechanical longitudinal coupling in the cochlea. It is not currently clear which of these types of longitudinal coupling are important to the proper function of the cochlea and which are not.

## Abbreviations

BM: Basilar membrane
CA: Cochlear amplifier
CEOAE: Click-evoked otoacoustic emission
CF: Characteristic frequency
CI: Cochlear implant
CM: Cochlea microphonic
CP: Cochlear partition
DOF: Degree of freedom
DP: Distortion product
DPOAE: Distortion product otoacoustic emission
FE: Finite element
FEA: finite element analysis
FFT: Fast Fourier transform
IFFT: Inverse fast Fourier transform
IHC: Inner hair cell
OAE: Otoacoustic emission
OC: Organ of Corti
ODE: Ordinary differential equation
OHC: Outer hair cell
OW: Oval window
PDE: Partial differential equation
PhPs: Phalangeal processes
RL: Reticular lamina
RM: Reissner's membrane
RW: Round window
SM: Scala medium
SOAE: Spontaneous acoustic emission
SPL: Sound pressure level
SV: Scala vestibuli
ST: Scala tympani
TM: Tectorial membrane
WFE: Wave finite element
WKB: Wentzel-Kramers-Brillouin approximation.
